# A multi-modal study on cerebrovascular dysfunction in cognitive decline of *de novo* Parkinson’s disease

**DOI:** 10.1016/j.nicl.2025.103836

**Published:** 2025-07-03

**Authors:** Hongwei Li, Xiali Shao, Jia Jia, Bingyi Wang, Jian Wang, Kai Liu, Jinhan Chen, Zhensen Chen, Lirong Jin, He Wang

**Affiliations:** a Institute of Science and Technology for Brain-inspired Intelligence, Fudan University, Shanghai, China; b Department of Radiology, Zhongshan Hospital, Fudan University, Shanghai, China; c Department of Neurology, Shanghai Xuhui Central Hospital, Shanghai, China; d Department of Neurology, Zhongshan-Xuhui Hospital, Fudan University, Shanghai, China; e Department of Neurology, Zhongshan Hospital, Fudan University, Shanghai, China; f MOE Key Laboratory of Computational Neuroscience and Brain-Inspired Intelligence, Fudan University, Shanghai, China; g Department of Radiology, Shanghai Fourth People’s Hospital Affiliated to Tongji University School of Medicine, Shanghai, China

**Keywords:** Parkinson’s disease, MRI, Cerebrovascular reactivity, Vascular function, Cognitive impairment

## Abstract

**Background:**

Vascular risk factors are increasingly implicated in Parkinson’s disease (PD), but the role of altered cerebrovascular dysfunction in early-stage PD remains unclear. Here, we investigated resting-state cerebrovascular reactivity (RS-CVR), cerebral blood flow (CBF), arterial morphological changes, and corresponding alterations in functional connectivity density (FCD) in *de novo* PD patients with different cognitive status.

**Methods:**

25 *de novo* PD patients with mild cognitive impairment (PD-MCI), 34 with normal cognition (PD-NC), and 48 healthy controls (HCs) underwent neuropsychological assessments and multimodal MRI. CBF derived from arterial spin labeling, RS-CVR and FCD generated from resting-state functional MRI and the arterial morphology extracted from the magnitude images of multi-echo gradient echo.

**Results:**

RS-CVR significantly decreased in PD patients, particularly in the left occipital gyrus and posterior cerebral artery (PCA) territories. Long-range FCD was reduced in the left inferior occipital gyrus in both PD-NC and PD-MCI compared to HCs (*p* = 0.005, *p* < 0.001). In PD-MCI, negative correlations between Stroop Color-Word Test time and RS-CVR in the distal right PCA (r = −0.71, *p*FDR = 0.030) and middle left PCA (r = −0.66, *p*FDR = 0.044) were observed. A significant correlation was found between decreased long-range FCD in the left inferior occipital gyrus and poorer Trail Making Test Part B performance (r = −0.63, *p*FDR = 0.029) in the PD-MCI. No significant differences in CBF, but significant dilation of the left PCA and compensatory CBF increases in the corresponding territory in PD-MCI were found (r = 0.57, *p*FDR = 0.023).

**Discussion:**

Microvascular dysfunction, rather than perfusion defects, might underlie early-stage of the *de novo* PD, especially in the patients with PD-MCI.

## Highlights

• Cerebrovascular dysfunction preferentially affects posterior circulation in early PD.
• CVR decline precedes CBF deficits in early PD.
• CVR reduction links cognitive deficits with reduced long-range FCD in early PD.
• Compensatory vasodilation preserves CBF in PD-MCI.

## 1 Introduction

Parkinson’s disease (PD) is a common progressive neurodegenerative disease. While α-synuclein-mediated neuronal loss is central to PD pathogenesis, emerging evidence suggests that the vascular alterations can aggravate the neurodegenerative process(Olaoye et al., 2025), exhibiting dynamic vascular pathological changes from pericyte activation (Elabi, 2021) and compensatory angiogenesis (Hansen, 2013) to the regression of blood vessels in the later stage(Paul and Elabi, 2022), yet their clinical significance in PD remains controversial.

Cerebral blood flow (CBF) measures capillary-level perfusion, and its alteration in PD exhibits heterogeneity across studies. Syrimi et al. identified posterior cortical hypoperfusion in non-demented patients with PD (Syrimi, 2017); but AI-Bachari et al. did not find a significant difference in CBF between PD patients and healthy controls (HCs) (Al-Bachari, 2014). Additionally, a previous report by Xiong et al. indicated that levodopa therapy had an impact on cerebral arterial morphologies and CBF in PD (Xiong, 2022). In addition to CBF, cerebrovascular reactivity (CVR), which partially reflects the integrity of the neurovascular unit, is also crucial for assessing microvascular functions (Kopczak, 2023). AI-Bachari et al and Pelizzari et al demonstrated that CVR in PD did not differ significantly from that of HCs (Al-Bachari, 2014). In contrast, Ryman et al. found reduced whole brain CVR in PD patients compared with HCs (Ryman, 2023). This discrepancy may be partly attributable to differences in scanning protocols; measuring methods and the characteristics of the subjects recruited across the studies. Furthermore, the concept of neurovascular coupling has been introduced (Iadecola, 2017). Alterations in the coupling between brain function and CBF (Zou, 2015), as well as synchronized changes between functional connectivity (FC) and CVR variations (Wang, 2024), have been observed in PD (Xie, 2023). Therefore, investigating various vascular function biomarkers, such as CVR and CBF, alongside assessments of arterial morphology and brain function changes in the early stages of *de novo* PD patients would be valuable, as this helps avoid potential confounding effects from medications on vascular function.

Over the past several decades, a growing selection of non-invasive techniques utilizing magnetic resonance imaging (MRI) has been developed for the assessment of cerebral hemodynamic parameters (Gauthier and Fan, 2019, Joshi et al., 2023, Lu et al., 2013). The 3D pseudo-continuous arterial spin labeling (PCASL) sequence has been validated as a robust MR method for the absolute quantification of CBF without any contrast agents (Alsop, 2015). The CVR measurement primarily relies on CO2 inhalation approaches using blood oxygen level dependent (BOLD)-functional MRI (fMRI) (Liu et al., 2019), but a post-processing method only using resting-state fMRI with specialized frequency band filtering has also been proposed (Liu, 2017), which does not require breath-hold modulation and offers new possibilities for clinical applications (Wang, 2024, Yeh, 2022, Ni, 2021). Magnetic resonance angiography (MRA) is a well-established technique that effectively captures the morphological information of large vessels (Edelman and Koktzoglou, 2019). However, the arterial morphologies, which represent an additional dimension of cerebral hemodynamic beyond perfusion, have often been overlooked and not fully utilized, particularly in the context of PD (Paul and Elabi, 2022, Xiong, 2022). To date, there has been limited research quantitatively assessing these vascular biomarkers simultaneously within the same PD cohort. Hence, the synchronous changes in brain vascular pathology in PD warrant in vivo verification using multi-modal MRI.

Thus, the aim of this study is to investigate the patterns of CVR, CBF, cerebral arterial morphologies and brain function alterations in the early drug-naïve PD patients using multi-modal MRI. Firstly, the PD patients were categorized into those with mild cognitive impairment (PD-MCI) and those with normal cognition (PD-NC), and group differences in above mentioned biomarkers were examined between these groups. Since cerebral vascular dysfunction was closely related with Alzheimer's disease and mild cognitive impairment (Cantin, 2011, Zhu et al., 2022); we subsequently determined whether the cerebral vascular function variations were associated with the cognitive decline in PD patients. Finally, based on these findings, we sought to elucidate the relationships between different biomarkers and their potential for differentiation of cognitive impairment in *de novo* PD patients. This study extends traditional unidimensional assessments of cerebrovascular function by providing novel in vivo evidence of integrated macro- and microvascular alterations during the early pathogenesis of Parkinson’s disease.

## 2 Materials and methods

### 2.1 Participants

This study was approved by the ethical committee of Zhongshan Hospital, Fudan University, and written informed consent was obtained from each subject. We recruited 59 *de novo* PD patients and 48 age and gender matched healthy control subjects. The PD patients were recruited from the movement disorders clinic at Zhongshan hospital. Inclusion criteria were (a) PD diagnosed by senior neurologists according to the Movement Disorder Society clinical diagnostic criteria for Parkinson's disease (Postuma, 2015); (b) Hoehn-Yahr 1–2, (c) education > 6 years. Exclusion criteria were (a) current or past use of anti-Parkinson medications, (b) a history of other neurological or psychiatric diseases or evidence of brain damage, (c) diagnosis of PD with dementia, (d) failure to complete primary school or the required scales and tests and (e) contraindications to MRI. Control subjects were recruited as volunteers from the community, with no history of neurological or psychiatric disorders, and all had completed at least 6 years of education. Neuropsychological evaluations were conducted for both control subjects and PD patients. PD-MCI was diagnosed using the level 2 criteria from the MDS Task Force 2012 (Goldman, 2013); defined as scores 1.5 standard deviations (SD) below the mean values derived from the community-based Shanghai Aging Study cohort established by Huashan Hospital (Ding, 2014). Impairment on at least two neuropsychological tests was required, either through two tests in one cognitive domain or one test in two different domains. PD patients not meeting the PD-MCI criteria were classified as PD-NC.

### 2.2 Clinical Examination

We compared clinical evaluations and performance on each neuropsychological test among the subjects, according to our previous study (Li, 2019). Briefly, Part III of the Unified Parkinson’s Disease Rating Scale (UPDRS III) and Hoehn and Yahr (H-Y) staging were used for motor function assessment. Neuropsychological evaluations including executive functions, attention/working memory, visuospatial functions, memory and language were performed with the following tests: Mini Mental State Examination (MMSE), Stroop Color and Word Test (SCWT), Trail Making Test-B (TMT-B), Symbol Digit Modalities Test (SDMT), Trail Making Test-A (TMT-A), Rey-Osterrieth Complex Figure Test (CFT), Clock-Drawing Test (CDT), Auditory Verbal Learning Test (AVLT), CFT-delayed recall, Animal Fluency Test (AFT), Boston Naming Test (BNT). 30-item Chinese version of the Geriatric Depression Scale (GDS) were also used.

### 2.3 Image acquisition

All MR data were acquired using a 3-Tesla MR unit (Discovery™ MR750, GE Healthcare, Milwaukee, WI). Resting-state fMRI images were acquired by using single-shot gradient-echo echo-planar imaging, covering the whole brain. The parameters of fMRI sequence were: TR = 2000 ms, TE = 30 ms, field of view = 24 cm, matrix size = 64 × 64, number of slices = 34, slice thickness = 4.0 mm, number of dynamic scans = 210. ASL images were acquired using a 3D PCASL sequence with background suppression and outward-direction spiral readout. The parameters of PCASL were as follow: TR = 4830 ms; TE = 10.5 ms; labeling duration = 1500 ms; post labeling delay (PLD) = 2025 ms; NEX = 3; flip angle = 155°; eight spiral arms with 512 points in each arm; bandwidth = 62.5 kHz. An additional proton density-weighted image for absolute CBF quantification used the same acquisition parameters. A 3D multi-echo gradient echo (mGRE) sequence was acquired, and the magnitude image of the shortest echo was used to perform the vessel segmentation. The mGRE scanning parameters were as follows: TR = 51.5 ms; number of echoes = 16; first TE = 2.9 ms; TE spacing = 3 ms; bandwidth = 62.5 kHz; flip angle = 12°; FOV = 22 cm; matrix size = 220 × 220; acceleration factor = 2. A high-resolution T1-weighted 3D acquisition for anatomical reference by using a brain volume imaging (BRAVO) sequence. The parameters of BRAVO sequence were: TR = 8.2 ms, TE = 3.2 ms, inversion time = 450 ms, flip angle = 12°, FOV = 240 mm × 240 mm, matrix = 256 × 256, slice thickness = 1.0 mm; no intersection gap; number of excitations = 1, number of slices = 136. Conventional MRI including T1-weighted, T2-weighted, fluid-attenuated inversion recovery (FLAIR) images and diffusion-weighted image sequences were obtained to exclude other pathological imaging findings that might interfere with further imaging assessment, such as acute infarction, moderate to severe small vessel disease and hippocampal atrophy.

### 2.4 BOLD fMRI analysis

**Fig. 1:**
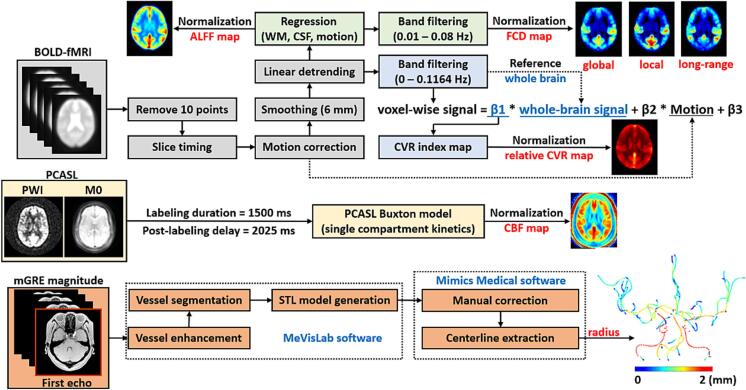
Overview of multi-modal MRI imaging analysis. Cerebrovascular reactivity (CVR), the amplitude of low-frequency fluctuations (ALFF), and functional connectivity density (FCD) are obtained through the post-processing of blood oxygen level dependent (BOLD)-functional MRI (fMRI). The perfusion-weighted image (PWI) and proton density image (M0) generated from pseudo-continuous arterial spin labeling (PCASL) are used for the absolute quantification of cerebral blood flow (CBF). The measurement of arterial radius is based on the magnitude of the first echo from a multi-echo GRE (mGRE) sequence after segmentation. All derived quantitative maps were normalized by the whole-brain mean values for the statistical analysis. All the quantitative maps shown here are average mean maps in the MNI space.

Resting-state BOLD fMRI analysis was conducted using the software Statistical Parametric Mapping (SPM) (University College London, UK) and in-house MATLAB R2024b (The MathWorks, Natick, MA) scripts. The pre-processing steps included removal of the first 10 volumes, slice-timing, motion correction, smoothing with an isotropic Gaussian kernel of 6 mm and linear detrending. For the resting-state CVR (RS-CVR) quantification, a frequency range of 0–0.1164 Hz was selected for the band filtering (Liu, 2022), which was found to provide the highest spatial correlation with CO2-inhalation CVR (Liu, 2021), and then the voxel-wise regression analysis was performed to generate a CVR index map, in which the whole brain signal was treated as an independent variable in the general linear model (Liu, 2017). The CVR index map was then normalized by the whole-brain mean value to obtain a relative CVR map, as depicted in Fig. 1.

For standard fMRI analysis, the pre-processed BOLD images were temporally filtered within the typical frequency range of 0.01–0.08 Hz (Fox, 2005); following the regression of white matter signal, ventricular signal and Friston 24 head motion parameters obtained from motion correction (Friston et al., 1996). The amplitude of low-frequency fluctuations (ALFF) was calculated by summing the frequency bins within the low-frequency band (0.01–0.08 Hz) of the power spectrum, calculated through a Fourier transform of the time-domain signal (Wu, 2008). Instead of performing conventional FC analysis, we chose to calculate functional connectivity density (FCD) (Tomasi and Volkow, 2012), a more quantitative indicator. The global FCD (gFCD) was defined as the total number of voxels with a Pearson correlation greater than 0.6 with the target voxel across the whole brain (Zhuo, 2017). The local FCD (lFCD) was determined by progressively including neighboring voxels with the correlation greater than 0.6 through a region-growing approach, ultimately yielding the voxel count of the final local cluster (Chen, 2015). The long-range FCD (lrFCD) was then derived as the difference between gFCD and lFCD (Li, 2019). For standardization purpose, the ALFF and FCD maps were further divided by the global mean values for subsequent statistical analysis.

### 2.5 CBF quantification

Perfusion maps were directly calculated according to PCASL one-compartment model (Alsop, 2015):

$$CBF=6000∙\frac{\lambda ∙e^{PLD/T_{1a}}}{2∙\alpha ∙\left(1-e^{-\tau /T_{1a}}\right)}∙\frac{\Delta M}{M_{0}}\left(mL/100g/min\right)$$

where λ=0.9mL/g is the brain/blood partition coefficient, ΔM and $M_{0}$ represent the signal intensity of perfusion difference image and proton density-weighted image, and τ is the labeling duration. $T_{1a}=1650ms$ is the longitudinal relaxation time of arterial blood, and α is the labeling efficiency, typically assumed to be 0.85. The quantitated CBF maps were normalized by dividing the subject’s global mean CBF value.

### 2.6 Arterial morphology

The mGRE magnitude images were used to measure arterial morphology. As shown in Fig. S1, vessel segmentation was performed on the shortest/first echo of the mGRE magnitude image, which was first interpolated to 0.86 mm isotropic resolution, using self-developed routines based on a specialized software MeVisLab (Mevis, Germany). The enhanced vascular structures were obtained by multiscale Hessian-based methods, followed by a region growing algorithm to create vessel masks, exporting STL files for morphology measurements. All segmentation results were visually checked and corrected manually by two neurologists, J Wang and XL Shao, who have over 5 years’ experience with neuroimaging interpretation. Finally, large proximal arterial radius was measured from vessel centerlines which were extracted using Mimics Medical 21.0 software (Materialize NV, Leuven, Belgium). For each vessel segment, the radius was determined by averaging measurements from at least five slices in a 3D viewer, which served as the representative radius value for that segment. Our MeVisLab configuration file for vessel segmentation, along with the STL files from two PD patients, can be accessed via: https://github.com/SpinEvo/pd_vessel_seg.

### 2.7 Statistical analysis

The RS-CVR, FCD, ALFF and CBF maps were warped into the Montreal Neurological Institute (MNI) space using each subject’s T1-weighted structural image as reference. These registered maps were segmented based on the third version of automated anatomical labeling (AAL3) atlas (Rolls et al., 2020) and a 18-ROI arterial territory template (Mutsaerts, 2015). The AAL3 brain atlas comprises 166 ROIs and the territory template covers the bilateral territories of proximal, middle and distal anterior cerebral artery (ACA), middle cerebral artery (MCA), and posterior cerebral artery (PCA). The mean regional values of each biomarker were calculated. Then the ROI-based statistical analyses were conducted in MATLAB. Two operators (J Wang and XL Shao) independently measured arterial morphological metrics in 25 PD-MCI patients to assess inter-rater reliability, using a two-way random effects model to calculate the intraclass correlation coefficient (ICC, for more details of the ICC analysis, see Supplementary Table S1) (Koo and Li, 2016), and the non-parametric Wilcoxon signed rank test was used to examine whether there were significant differences between the two operators' measurements.

Demographic characteristics, clinical scales, and all imaging metrics, including RS-CVR, gFCD, lFCD, lrFCD, ALFF, CBF and arterial radius, were compared among the three groups using the non-parametric Kruskal-Wallis test. Separate FDR multiple comparison corrections using the linear step-up procedure (Benjamini and Hochberg, 1995) were performed for each individual metric. After FDR correction; the remaining significant results were further examined using Fisher's least significant difference procedure to assess the differences between the two groups (Meier, 2006). Given the inherent property of absolute quantifiability in CBF using PCASL, the non-normalized whole-brain CBF changes were analyzed separately and compared across the three groups.

Moreover, to investigate whether these vascular function changes were associated with cognitive impairment, bivariate Spearman correlation were conducted within each group, focusing only on the significant difference regions of each imaging metrics identified from the previous comparisons. The FDR correction procedure was performed exclusively on the *p* values derived from bivariate correlations between a given imaging metric in a specific subject group and a particular atlas against all neuropsychological evaluations (CFT-delay, AVLT, BNT, AFT, SDMT, TMT-A, CFT, CDT, CWT-time, CWT-right, TMT-B, MMSE). Partial correlation analyses were further performed on the significant correlation pairs after controlling for covariates, gender, education, age, UPDRS-III scores and GDS scores. In addition, we also analyzed the correlation between significantly altered arterial radius and CBF in the corresponding vascular territories and brain regions across the three groups to explore their potential synchronized changes. FDR correction was independently applied to all *p* values within each group. The significance level was set at *p* = 0.05.

Finally, we aimed to assess the predictive ability of all the significantly different imaging biomarkers, from the Kruskal-Wallis test mentioned above, in distinguishing between PD-NC and PD-MCI in *de novo* PD patients, in order to identify the most discriminative indicators. The least absolute shrinkage and selection operator (LASSO) algorithm was first used to fit the logistic regression model, which could further identify the best predictors and avoid overfitting (Ambler et al., 2002). More specifically, we employed 10-fold cross-validation with L1 regularization to select the optimal regularization strength that minimized the mean squared error (MSE). Receiver operating characteristic (ROC) curves were generated to evaluate the individual diagnostic performance of these LASSO-selected biomarkers, and the area under the curve (AUC) was calculated.

## 3 Results

### 3.1 Demographic and clinical characteristics

The participants’ demographic and clinical characteristics were summarized in Table 1.

**Table 1:** Clinical and cognitive features in PD-NC patients, PD-MCI patients, and control subjects.

	**PD-MCI**	**PD-NC**	**Controls**	**Kruskal-Wallis**	**PD-MCI vs PD-NC**	**PD-MCI vs Controls**	**PD-NC vs Controls**
	**(n=25)**	**(n=34)**	**(n=48)**	***P* values**			
Age(y)	67.88 ± 5.82	62.65 ± 9.81	64.08 ± 7.67	0.06			
Sex(M/F)	12/13	16/18	21/27	0.927			
GDS-30 scores	11.76 ± 5.13	9.32 ± 6.45	6.10 ± 5.00	<0.001			
H&Y	1.44 ± 0.58	1.25 ± 0.43		0			
UPDRS III score	19.76 ± 8.89	15.12 ± 7.10		0.03			
Disease duration(month)	14.78 ± 8.90	19.79 ± 16.01		0.166			
**Cognitive characteristics**							
MMSE	27.80 ± 1.67	28.88 ± 1.37	29.06 ± 1.14	0.001	0.003	<0.001	0.552
**Memory**							
AVLT-T	19.92 ± 6.16	30.85 ± 10.91	31.79 ± 7.90	<0.001	<0.001	<0.001	0.63
CFT-delay recall	10.60 ± 3.84	16.71 ± 6.90	16.59 ± 6.36	0.001	<0.001	<0.001	0.935
**Visuospatial function**							
CFT copy	32.78 ± 2.71	31.29 ± 4.29	31.48 ± 3.63	0.027			
CDT	8.38 ± 1.50	9.29 ± 0.76	9.35 ± 0.73	<0.001	0.001	<0.001	0.782
**Language**							
AFT	13.50 ± 3.66	19.29 ± 5.48	19.00 ± 5.82	<0.001	<0.001	<0.001	0.805
BNT	21.52 ± 4.40	25.85 ± 2.06	25.9 ± 2.87	<0.001	<0.001	<0.001	0.951
**Attention and working memory**							
SDMT	24.24 ± 9.62	40.03 ± 9.25	45.08 ± 10.94	<0.001	<0.001	<0.001	0.028
TMT-A	96.08 ± 37.63	57.85 ± 16.46	51.92 ± 19.15	<0.001	<0.001	<0.001	0.27
**Executive function**							
CWT-C-time	113.68 ± 57.57	79.29 ± 18.41	69.44 ± 18.95	<0.001	<0.001	<0.001	0.189
CWT-C-right	43.60 ± 5.32	46.65 ± 3.33	47.83 ± 2.32	<0.001	0.001	<0.001	0.138
TMT-B	204.26 ± 63.37	131.09 ± 36.57	118.94 ± 41.64	<0.001	<0.001	<0.001	0.239

Abbreviations: PD, Parkinson’s disease; M, male; F, female; H&Y, Hoehn and Yahr stage; UPDRS-III, Unified Parkinson’s Disease Rating Scale; GDS, 30-item Chinese version of the Geriatric Depression Scale; MMSE, Mini Mental State Examination; AVLT, Auditory Verbal Learning Test; CFT, Rey-Osterrieth Complex Figure Test; CDT, Clock Drawing Test; AFT, Animal Fluency Test; BNT, 30-item Boston Naming Test; SDMT, Symbol Digit Modality Test; TMT-A, Trail Making Test A; TMT-B, Trail Making Test B; SCWT, Stroop Color-Word Test.

### 3.2 Group comparison of MR metrics

**Fig. 2:**
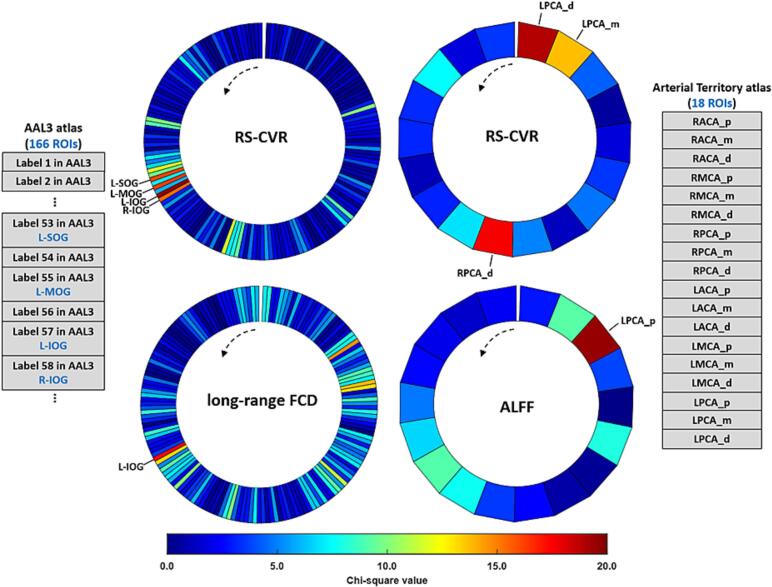
Comparison of quantitative maps among healthy controls (HCs), Parkinson's disease with normal cognition (PD-NC), and Parkinson's disease with mild cognitive impairment (PD-MCI) groups. The AAL3 atlas contained 166 regions of interest (ROIs), while the arterial territory atlas comprised 18 ROIs that were used to extract imaging metrics. All territories in the arterial territory atlas were labeled in the rightmost panel of the figure. The first column displayed significant findings from the AAL3, and the second column presented significant results derived from the territory atlas. All ROIs were arranged in counterclockwise order. Significant differences in all imaging metrics were restricted to the occipital lobe: specifically, the left superior, middle, and inferior occipital gyri (L-SOG/L-MOG/L-IOG) and right inferior occipital gyrus (R-IOG) in the AAL3 atlas, and exclusively to posterior cerebral artery (PCA) territories, including proximal, middle, and distal territories of the left PCA (LPCA) and distal territory of the right PCA (RPCA) in the arterial territory template. The color scale represented Chi-square values from the Kruskal-Wallis test. p, proximal territory; m, middle territory; d, distal territory.

As shown in Fig. 2, significant differences in all imaging metrics were exclusively observed in the occipital lobe, specifically the left superior occipital gyrus (L-SOG), left middle occipital gyrus (L-MOG), left inferior occipital gyrus (L-IOG), and right inferior occipital gyrus (R-IOG) under the AAL3 atlas, and solely within PCA territories, including proximal, middle and distal territories of the left posterior cerebral artery (LPCA) and distal territory of the right posterior cerebral artery (RPCA) in the arterial territory template.

**Fig. 3:**
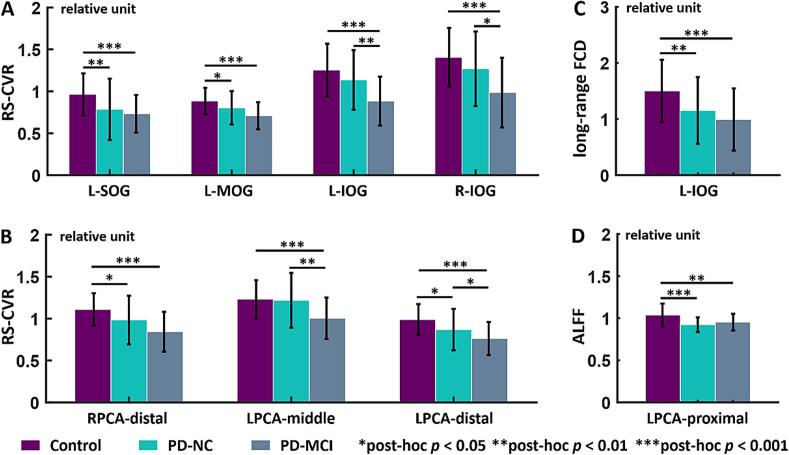
Quantitative maps showed significant differences among healthy controls (HCs), Parkinson's disease with normal cognition (PD-NC), and Parkinson's disease with mild cognitive impairment (PD-MCI) groups. (A) Resting-state cerebrovascular reactivity (RS-CVR) in the left superior occipital gyrus (L-SOG), middle occipital gyrus (L-MOG) was significantly lower in PD-NC and PD-MCI groups compared to the HCs. In the left and right inferior occipital gyrus (L-IOG, R-IOG), PD-MCI showed significantly lower RS-CVR than the HCs and PD-NC groups. (B) RS-CVR in the distal territory of right posterior cerebral artery (RPCA) was significantly lower in the PD groups compared to the HCs. RS-CVR in the middle territory of left posterior cerebral artery (LPCA) was the lowest in PD-MCI groups. RS-CVR in the distal territory of the LPCA showed differences among the three groups, with the PD-MCI having the lowest level, followed by the PD-NC groups. (C) Long-range functional connectivity density (FCD) was significantly reduced in the L-IOG in PD-NC and PD-MCI groups compared to the HCs. (D) The amplitude of low-frequency fluctuations (ALFF) in the proximal territory of LPCA was significantly lower in PD-NC and PD-MCI groups compared to the HCs. All data were shown in mean ± STD. The non-parametric Kruskal-Wallis test was used for comparisons among the three groups, only the significant results surviving from the false discovery rate (FDR) correction were shown here. Significance of post-hoc analysis: * *p* < 0.05, ** *p* < 0.01, *** *p* < 0.001.

Significant RS-CVR differences among the three groups were observed in the L-SOG (Chi-square = 16.07, df = 2, *p*FDR = 0.012), L-MOG (Chi-square = 16.25, df = 2, *p*FDR = 0.012), L-IOG (Chi-square = 19.60, df = 2, *p*FDR = 0.007), and R-IOG (Chi-square = 15.45 df = 2, *p*FDR = 0.014). As shown in Fig. 3A, the post-hoc analysis revealed significantly lower RS-CVR in the L-SOG and L-MOG for both the PD-NC and PD-MCI groups compared to the HCs (L-SOG: *p* = 0.0017, *p* < 0.001; L-MOG: *p* = 0.0279, *p* < 0.001, respectively), with no significant differences observed between the two PD groups in these two gyri. In the L-IOG, PD-MCI showed significantly lower RS-CVR than the HCs and PD-NC groups (*p* < 0.001, *p* = 0.0053, respectively). Similarly in the R-IOG, significantly lower RS-CVR was observed in PD-MCI compared to HCs and PD-NC (p < 0.001, p = 0.0210, respectively). Differences in RS-CVR in the vascular territories were observed in the distal territory of RPCA (Chi-square = 17.64, df = 2, *p*FDR = 0.009), middle territory of LPCA (Chi-square = 13.70, df = 2, *p*FDR = 0.028) and distal territory of LPCA (Chi-square = 18.98, df = 2, *p*FDR = 0.007). As shown in Fig. 3B, RS-CVR in the distal territory of RPCA was significantly lower in the PD-NC and PD-MCI compared to the HCs (*p* = 0.0150, *p* < 0.001, respectively). In the middle territory of LPCA, PD-MCI showed significantly lower RS-CVR than the HCs and PD-NC groups (*p* < 0.001, *p* = 0.0070, respectively). RS-CVR in the distal territory of the LPCA showed differences among the three groups, with the PD-MCI having the lowest level, followed by the PD-NC group (HCs vs. PD-NC: *p* = 0.0239; HCs vs. PD-MCI: *p* < 0.001; PD-NC vs. PD-MCI: *p* = 0.0353).

However, there were no significant differences in any cortical or territory region for CBF, global FCD, and local FCD after FDR correction. The non-normalized global whole-brain CBF was 46.50 ± 6.92 mL/100 g/min in HCs, 47.18 ± 9.66 mL/100 g/min in the PD-NC group, and 43.23 ± 8.24 mL/100 g/min in the PD-MCI group, and also showed no significant group differences (Chi-sq = 5.1677, df = 2, *p* = 0.0755). Long-range FCD was only significantly reduced in the L-IOG (Chi-square = 17.28, df = 2, *p*FDR = 0.033) in PD-NC and PD-MCI groups compared to the HCs (*p* = 0.0052, *p* < 0.001, respectively, Fig. 3C). The ALFF differences was only observed in the proximal territory of LPCA (Chi-square = 19.50, df = 2, *p*FDR = 0.011), as shown in Fig. 3D, with lower ALFF in PD-NC and PD-MCI groups compared to the HCs (*p* < 0.001, *p* = 0.0083, respectively). Furthermore, Table 2 showed that significant arterial radius dilation of the P2 segment of the LPCA (Chi-square = 11.79, df = 2, *p*FDR = 0.035) was observed in the PD-NC and PD-MCI groups compared to the HCs (*p* = 0.0022, *p* = 0.0101, respectively). More details of the Kruskal-Wallis tests for all imaging metrics were provided in supplementary Tables S2–S13). Table S1 illustrated that intra-rater reliability exhibited relatively high and significant consistency, with no differences in the arterial radius measurements obtained by either operator.

**Table 2:** Cerebrovascular morphology metrics (arterial radius) in PD-NC patients, PD-MCI patients and controls.

**Morphology Metrics** **Mean (SD), unit *mm***	**Controls** **(n = 48)**	**PD-NC** **(n = 34)**	**PD-MCI** **(n = 25)**	***p* value**	**FDR Corrected** ***p* value**
RICA radius	2.31 (0.22)	2.37 (0.26)	2.48 (0.22)	0.029	0.094
LICA radius	2.36 (0.25)	2.34 (0.26)	2.51 (0.30)	0.130	0.241
RACA-A1 radius	1.38 (0.17)	1.43 (0.12)	1.42 (0.15)	0.304	0.360
LACA-A1 radius	1.39 (0.15)	1.44 (0.16)	1.45 (0.15)	0.160	0.261
RMCA-M1 radius	1.65 (0.11)	1.74 (0.14)	1.70 (0.12)	**0.018**	0.079
LMCA-M1 radius	1.60 (0.12)	1.69 (0.15)	1.68 (0.14)	**0.010**	0.064
RPCA-P1 radius	1.45 (0.13)	1.40 (0.21)	1.50 (0.17)	0.120	0.242
LPCA-P1 radius	1.45 (0.18)	1.47 (0.13)	1.52 (0.17)	0.081	0.211
RPCA-P2 radius	1.39 (0.10)	1.38 (0.13)	1.41 (0.21)	0.864	0.864
**LPCA-P2 radius**	1.31 (0.14)	1.41 (0.09)	1.40 (0.17)	**0.003**	**0.035**
BA radius	1.69 (0.22)	1.72 (0.23)	1.77 (0.20)	0.202	0.292

Significant statistical analysis results were shown in bold. The non-parametric Kruskal-Wallis test was used for comparisons among the three groups, with *p*-values displayed in the last two columns. All data were shown in mean ± SEM. Radius unit: mm; ICA: internal carotid arteries; MCA: middle cerebral arteries; ACA: anterior cerebral arteries; PCA: posterior cerebral arteries; BA: basilar arteries.

### 3.3 Distinct relationship between cognitive test scores and MR metrics

Bivariate Spearman correlation was used between imaging biomarkers surviving from the FDR correction in the above Kruskal-Wallis test and all neuropsychological evaluations in our study cohorts. As shown in Figs. S2 and S3, significant correlations were only found in the PD-MCI group after FDR correction. Regarding AAL3 brain regions, the only significant correlation was a negative association between long-range FCD in L-IOG and TMT-B (*r* = −0.6301, *p*FDR = 0.0287). For arterial territories, CWT-time demonstrated significant negative correlations with RS-CVR in the distal territory of the RPCA (*r* = −0.7091, *p*FDR = 0.0304) and the middle territory of the LPCA (*r* = −0.6634, *p*FDR = 0.0440). Additionally, a positive correlation was observed between ALFF in the proximal territory of the LPCA and TMT-A (*r* = 0.6538, *p*FDR = 0.0359).

**Fig. 4:**
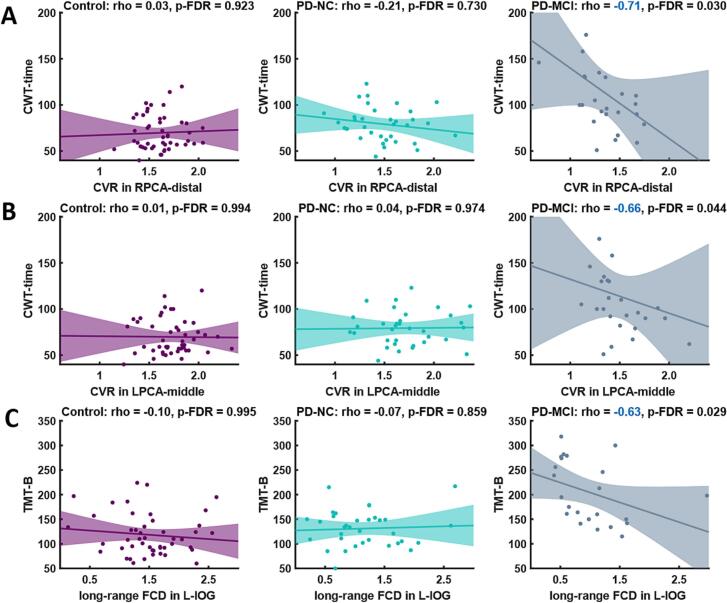
Significant correlation between quantitative maps and neuropsychology evaluations. (A, B) In PD-MCI group, a negative association between time of Stroop Color-Word Test (CWT-time) and cerebrovascular reactivity (CVR) in the distal territory of right posterior cerebral artery (PCA) and the middle territory of left PCA. (C) In PD-MCI group, a significant correlation was only found between decreasing long-range functional connectivity density (FCD) in the left inferior occipital gyrus (IOG) and poorer performance on the Trail Making Test B (TMT-B). The shaded areas indicated the 95% confidence interval for the fitted regression curve.

In the subsequent partial correlation analysis, the significant correlation between decreasing long-range FCD of L-IOG and poorer performance on the TMT-B (*r* = −0.7207, *p* = 0.0036) were retained in PD-MCI group after controlling for age, gender, education, UPDRS-III scores and GDS scores. The negative associations between CWT-time and RS-CVR in the distal territory of RPCA (*r* = −0.6455, *p* = 0.0127) and middle territory of LPCA (*r* = −0.7443, *p* = 0.0023) remained statistically significant in the PD-MCI group following covariates regression. The bivariate correlation between ALFF and CWT-time became non-significant (*r* = 0.2751, *p* = 0.3412) after adjusting for covariates. Correlations that remained significant after partial correlation analysis were presented in Fig. 4, with the bivariate correlation *r* values and FDR-corrected *p*-values displayed.

### 3.4 Association between arterial radius and CBF

**Fig. 5:**
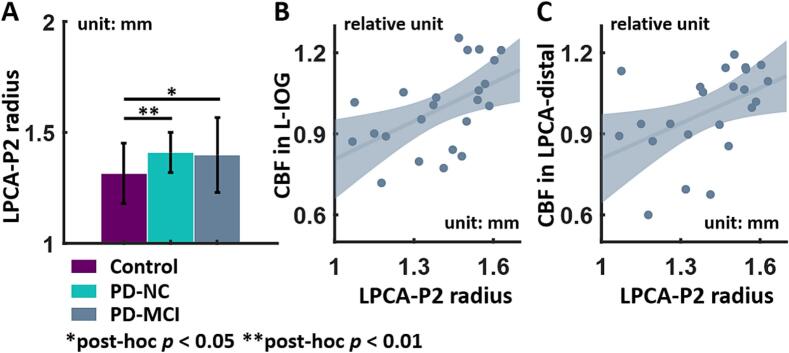
Synchronous changes between arterial radius and cerebral blood flow (CBF). (A) The radius of the P2 segment of left posterior cerebral artery (LPCA) was significantly dilated in PD groups. Data were shown in mean ± STD. (B, C) An increase in CBF within the left inferior occipital gyrus (L-IOG) and distal territory of the LPCA was observed exclusively in the PD-MCI group, correlating with the P2 segment of the LPCA arterial radius dilation. Noted that there was no significant CBF differences among the three groups. The shaded areas indicated the 95 % confidence interval for the fitted regression curve. Significance of post-hoc analysis: * *p* < 0.05, ** *p* < 0.01, *** *p* < 0.001.

We observed an increase in the arterial radius of the P2 segment of the LPCA in the PD groups, but there were no significant differences in CBF among the three groups. Since the occipital lobe was primarily supplied by the posterior cerebral arteries, CBF values for the significant brain regions previously identified — the left superior occipital gyrus, the left middle occipital gyrus, and the left inferior occipital gyrus, as well as the proximal, middle, and distal territories of the LPCA — were extracted and correlated with the LPCA-P2 arterial radius in each group. As shown in Fig. 5, only the CBF of the L-IOG (*r* = 0.5702, *p*FDR = 0.0231) and the distal territory of the LPCA (*r* = 0.5474, *p*FDR = 0.0231) showed significant positive correlations with the radius, which was only observed in the PD-MCI group. The results indicated that the increased CBF arising from arterial dilation might be pronounced only in the PD-MCI group.

### 3.5 Logistic regression and ROC analysis

The RS-CVR in L-SOG, L-MOG, L-IOG, R-IOG, RPCA distal territory, LPCA middle and distal territories, long-range FCD in L-IOG, ALFF in LPCA proximal territory and LPCA-P2 arterial radius were included in the logistic regression model. The minimum cross-validated MSE was achieved at a regularization parameter value of 0.0573. The best predictors determined through LASSO were RS-CVR in L-IOG, RS-CVR in the LPCA middle territory and ALFF in the LPCA proximal territory. The predictive ability to distinguish between the PD-NC and PD-MCI groups was illustrated by the individual ROC curves for each predictor presented in Fig. S4. The RS-CVR in the L-IOG achieved the highest AUC of 0.73 (0.58–0.86), which was almost comparable to the combination of all three predictors, with AUC of 0.75 (0.62–0.88).

## 4 Discussion and conclusions

In this study, we revealed significant regional RS-CVR reductions in drug-naïve PD-MCI and PD-NC patients compared to HCs, most prominently in the left occipital gyrus, while no significant CBF changes were observed across the three groups, suggesting that cerebrovascular dysfunction rather than perfusion defect might be involved in the early stage of the disease. Over the past two decades, the Vascular Hypothesis of Alzheimer's Disease (VHAD) has been proposed, positing that neurovascular unit dysfunction might be one of mixed pathologies (Eisenmenger, 2023). Numerous neuroimaging studies offer the evidences of vascular involvement in AD (Liu, 2024, van der Kleij, 2018, Aslanyan, 2024). In contrast, vascular contributions to PD pathophysiology, especially in PD with cognitive impairment, remain underexplored. Although vascular co-pathology may coexist with PD neurodegeneration (Elabi, 2021, Paul and Elabi, 2022), in vivo evidence remains scarce. Therefore, we implemented a multimodal MRI framework to characterize macrovascular and microvascular changes in early PD, while simultaneously evaluating FCD and cognitive status through standardized neuropsychological assessments.

CVR paradigms measured the ability of the brain vasculature in response to a vasoactive stimulus, and tightly couple with both intrinsic brain factors systemic factors (Carr et al., 2021, Schaeffer and Iadecola, 2021). The RS-CVR used in this study, while not entirely equivalent to classical CVR measures, has been shown to exhibit high spatial similarity with CVR measurements obtained via CO2 inhalation (Liu, 2021). In a recent review paper, Ryman and colleagues suggested CVR has also been to play a role in glymphatic clearance of α-Synuclein (Ryman, 2024). A previous report by van der Horn et al. proposed PD related CVR pattern characterized by relatively increased CVR latency in basal ganglia, sensorimotor cortex, supplementary motor area, thalamus and visual cortex, as well as decreased latency in the cerebral white matter, compared with healthy controls (van der Horn, 2024). Our results partly consisted with above-mentioned study that RS-CVR reduction might be obvious in posterior cortex. Our study recruited only drug-naïve PD patients of early stage, possibly explaining the differences between the two studies. The two studies also used different quantification and analytic methods in terms of the BOLD MRI results. Nonetheless, our results still indicated that the RS-CVR alteration occurred prior to CBF changes, and might serve as an early biomarker of PD.

Additionally, we found that the RS-CVR of the left and right IOG in PD-MCI patients was significantly lower than that of PD-NC and control subjects, suggesting that RS-CVR might associated with PD clinical phenotypes. Our results also showed that RS-CVR in the left IOG as the biomarker with the highest discriminative power for distinguishing PD-MCI from PD-NC, achieving an AUC of 0.73. The mechanism underlying cognitive impairment in PD is still unclear, although concomitant proteinopathies, neurotransmitter deficits, genetics and cerebrovascular dysfunction have been proposed to contribute (Ryman, 2024, Aarsland, 2021). With the development of neuroimaging technology, more studies investigated cerebral vascular changes such as white matter hyperintensity burden (Carvalho de Abreu et al., 2023); perivascular spaces (Chen, 2022, Park, 2019), and cerebral perfusion (Arslan, 2020, Wang, 2022) might be associated with cognitive impairment in PD or contributes to progression of cognitive decline. Our current finding combined with our previous report (Wang, 2024) suggested potentially reflecting cognitive reserve related to RS-CVR.

Moreover, synchronizing changes with RS-CVR alternation, long-range FCD in the left IOG was reduced in the PD group, most notably in patients with MCI, with a negative association with TMT-B time. This finding further confirmed the relationship among cerebrovascular dysfunction, brain activities and cognition (Huang, 2023). Functional connectivity density derived from resting state BOLD images, is an important indicator for brain region connections (Rubinov and Sporns, 2010). Our result was partly in accordance with previous study showing widespread decreased long-range FCD of major cortical and subcortical areas in the patients with PD-MCI (Baggio, 2014).

Interestingly, our previous work has shown that RS-CVR in the left IOG was not only involved in the progression of cognitive impairment in PD patients but also exhibited a trend of decreasing functional connectivity relative to the left IOG, with this reduction spreading from the posterior to the anterior regions of the brain, particularly the frontal lobe (Wang, 2024). This pattern aligns with the concept of long-range FCD, suggesting that the RS-CVR reduction might also contribute to the onset of cognitive deficits, partially mediated by decreased long-range FCD.

In order to identify RS-CVR changes from macro-vascular scale, we then adopted RS-CVR maps in bilateral anterior, middle, and posterior cerebral arteries and investigated the relationship between neuropsychological evaluations and RS-CVR in associated vascular territories. Our results demonstrated that the RS-CVR in the distal and middle territories of left PCA in PD-MCI patients was significantly lower than that of PD-NC patients and controls. The RS-CVR for PD-NC patients was intermediate between controls and PD-MCI patients. It would be interesting to test in future studies whether PCA-territory RS-CVR could predict cognitive decline in PD. We noted that of the 12 neuropsychology evaluations, only time of completing SCWT was correlated with RS-CVR from segment of PCA in PD-MCI group. SCWT, an executive function test might be associated with alerting, speed of visual search, orienting, working memory and so on (Treisman and Fearnley, 1969, Periáñez et al., 2021). Cognitive impairment in PD initially was characterized by executive and visuospatial dysfunctions (Weintraub et al., 2018), partly accompanied with posterior cortical deficits (Devignes, 2021). Some studies reported the correlation between CVR reduction and cognitive decline in adults with risk of cognitive decline (Kim, 2021), and the patients with mild cognitive impairment or Alzheimer’s disease (Richiardi, 2015). Our findings implied impaired RS-CVR from the area supplied by the posterior cerebral artery might partly contribute to cognitive deficits in PD. Further longitudinal studies involving larger sample sizes and extended follow-up durations are necessary to evaluate the potential of baseline RS-CVR as a predictor for the conversion from PD-MCI to PD dementia.

Besides, dilation of the P2 segment of the left PCA was observed in both PD-MCI and PD-NC groups relative to HCs, which might represent a potential compensatory process. Alternation of arterial morphology in PD was rarely reported. Xiong et al. showed significantly lower CBF and shortened total artery length at medication off state, and CBF elevated corresponding with dilation of radius of proximal arteries after taking Levodopa (Xiong, 2022). By recruiting only *de novo* early-stage PD patients, the potential confounding effects of medication or long disease progression was bypassed. Also of note, there were no significant changes in CBF among three groups, and artery radius change significantly related to CBF change in corresponding territories. This result suggested normal cerebral perfusion might an adaptive compensatory mechanism responding to dilating arteries. In the future, the development of magnetic resonance fingerprinting might enable micro cerebrovascular metrics including quantitative CBV, vessel size and CVR in neurodegenerative disease (Wheeler et al., 2023).

In our study, the relatively widespread alterations in RS-CVR within the occipital lobe, occurring without concomitant changes in CBF and accompanied by dilation of PCA radius, might potentially indicate the presence of a compensatory mechanism mediated by cerebral autoregulation (Strandgaard and Paulson, 1992), which involved pronounced endothelial vasodilatation as a key component of mechanoregulation in the cerebral vasculature (Traystman, 1997), to maintain perfusion in early-stage PD-MCI. CVR, reflecting the responsiveness of brain vasculature under challenge rather than direct autoregulation (Lavi et al., 2003), has been observed as impaired alongside preserved CBF in MCI studies (Glodzik et al., 2013), suggesting it might serve as an early biomarker of vascular dysfunction, although this remains speculative and requires further validation. Long-range FCD and ALFF demonstrated significant differences, but as shown in Fig. 3, these primarily distinguished PD from HCs, whereas PD-NC versus PD-MCI differences were predominantly manifested in RS-CVR.

Subcortical pathology was recognized as a hallmark feature of PD, particularly evident in molecular imaging (Mitchell, 2021). However, molecular imaging and MRI are distinct modalities, and the spatial patterns of alterations also differed across MR sequences. Prior studies demonstrated that spatial dissociation between structural changes and vascular function patterns might exist in both cerebrovascular disorders (Anazodo, 2016) and normal aging (Chen et al., 2011). Similar dissociation was considered likely in PD, as evidenced by previous findings of structural atrophy in the left caudate nucleus alongside significantly reduced perfusion in the left precuneus in PD-MCI patients (Jia et al., 2019). Furthermore, another investigation revealed that even in advanced PD with dementia, significant CBF alterations primarily occurred in cortical regions, particularly within posterior circulation territories, while subcortical perfusion remained preserved (Melzer, 2011). These collective observations provided support for the notion that early vascular functional changes in PD might emerge earlier in cortical areas.

This study employed techniques that avoid the use of contrast agents or CO2 stimulation, making them well-suited for elderly patients with PD. The significance of multimodal MRI lies in its capacity to offer comprehensive, and also comparable information provided by different sequences and post-processing methods. Previous studies have demonstrated the repeatability of RS-CVR (Wang, 2024, Liu, 2023), FCD (Tomasi et al., 2016, Xiuchao, 2015) measured with rsfMRI, and CBF assessed with PCASL (Wu et al., 2014), while we have also validated the reliability of radius measurements using our own data. Thus, several imaging metrics, RS-CVR, CBF, FCD and arterial radius were included, to maximize the full potential of the data, capturing the entire process of cerebral hemodynamics from macro to microvascular function.

We did not acquire MRA data, instead we used the first echo of the mGRE sequence for the vessel segmentation. This sequence's readout pattern was similar to the commonly used TOF MRA sequences for the brain (Price et al., 1992), and the first echo provided strong bright arterial signal with a very short echo time of 2.9 ms, without the concern of susceptibility-induced blooming observed in mGRE images. Intra-rater reliability showed relatively high overall consistency between the two operators, with values exceeding 0.65; however, the consistency for one vessel, RPCA-P2, was only 0.45. The lower ICC observed in RPCA-P2 might partially contribute to the laterality observed in arterial radius findings, although CVR results based on the territory template also demonstrated leftward lateralization. This discrepancy primarily arose from our manual selection of an average radius for each arterial branch with at least five slices, rather than calculating the radius at every point along the centerline. Defining branch points of the PCA-P2 segment proved challenging due to difficulties in segmenting these small-diameter vessels, which might introduce some positional measurement bias along this relatively long vessel segment between different operators. To accurately define the start and end points of each arterial branch, high-quality MRA data and detailed vessel labeling were needed (Chen, 2019), making the process time-consuming and labor-intensive—efforts that were not strictly essential for measuring radius.

In addition to small sample size, our study also has other limitations. The patients recruited in this study had not all underwent dopamine transporter photon emission scanning. In this study, patients with an expert-confirmed clinical diagnosis of PD at baseline were followed up for 3 years, to minimize the potential possibility of misdiagnosis. Also, we did not assess CVR through resting-state fMRI with hypercapnic stimulus. Future studies, ideally from multiple centers and with more patients recruited, fMRI scans with CO2 challenge would be essential for verify the findings of current study.

In conclusion, our results contribute to a better understanding of the relationship between neurovascular unit integrity, cerebral artery morphology and arterial territory perfusion in *de novo* early PD. In the future, we should identify the risk factors that could be managed to preserve CVR in an effort to prevent cognitive decline in PD.

## Funding statement

H.W. is supported by National Key R&D Program of China (2023YFF1204804), National Natural Science Foundation of China (No. 82271956, No. 62331021) and Shanghai Municipal Science and Technology Explorer Project (No. 23TS1400500). Z.C. is supported by Natural Science Foundation of Shanghai (22ZR1403900) and National Natural Science Foundation of China (No. 82302156). J.J. receives research support from Medical Research Project in Xuhui District, Shanghai (SHXH202234) and 2024–2025 Xuhui District Guangqi Talent Action Plan “Guangqi Famous Doctors” Cultivation Project. The funder played no role in study design, data collection, analysis and interpretation of data, or the writing of this manuscript.

## CRediT authorship contribution statement

**Hongwei Li:** Writing – review & editing, Writing – original draft, Validation, Software, Methodology, Investigation, Formal analysis, Conceptualization. **Xiali Shao:** Writing – review & editing, Validation, Resources, Formal analysis, Data curation. **Jia Jia:** Writing – review & editing, Writing – original draft, Resources, Investigation, Data curation. **Bingyi Wang:** Software, Methodology. **Jian Wang:** Validation, Resources, Methodology, Investigation, Data curation. **Kai Liu:** Data curation. **Jinhan Chen:** Methodology. **Zhensen Chen:** Supervision, Project administration. **Lirong Jin:** Writing – review & editing, Writing – original draft, Validation, Supervision, Resources, Project administration, Methodology, Investigation, Data curation, Conceptualization. **He Wang:** Writing – review & editing, Supervision, Resources, Project administration, Funding acquisition.

## Data Availability

A portion of the extracted image data and corresponding statistical results are accessible via the following link: https://zenodo.org/records/15620135.
