# Supplementary material for: A multi-modal study on cerebrovascular dysfunction in cognitive decline of de novo Parkinson’s disease
Source: Neuroimage Clin. 2025 Jul 3;48:103836. doi: 10.1016/j.nicl.2025.103836 (PMC12275129; doi:10.1016/j.nicl.2025.103836)
Supplement: Supplementary Data 1 [file mmc1.docx]

**FIG. S1.** Artery morphological parameters were extracted from the first echo magnitude of multi-echo gradient echo (mGRE) sequence. Flowchart of semi-automated analysis of arterial morphology, including vessel enhancement, segmentation, arterial centerline extraction, and radius measurement.


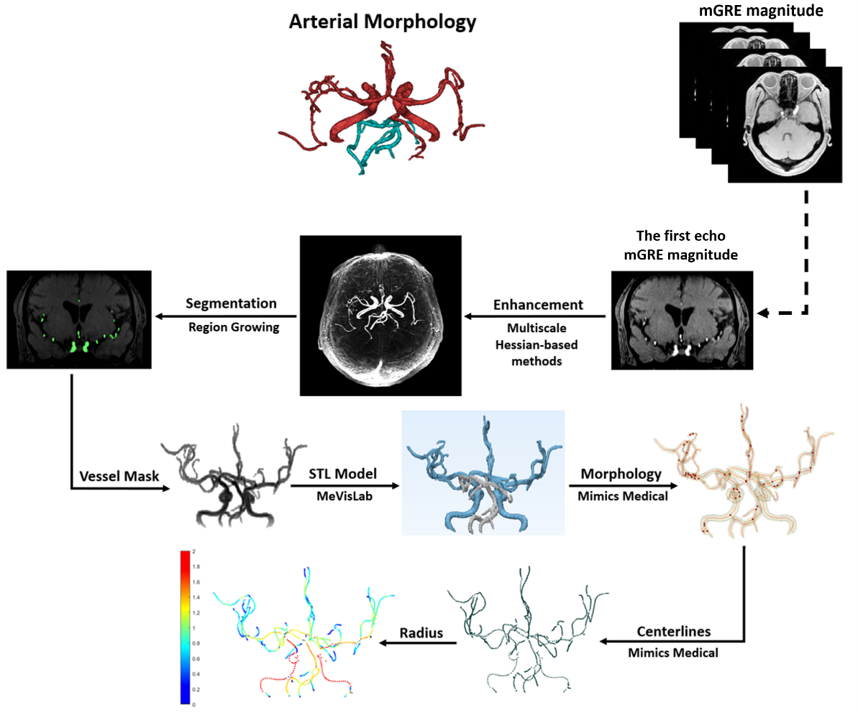


**FIG. S2.** Bivariate Spearman correlation analysis between imaging metrics extracted from the AAL3 atlas and 12 neuropsychological evaluations. The only significant correlation was a negative association between long-range FCD in L-IOG and TMT-B (*r* = -0.6301, *p*FDR = 0.0287). Abbreviations: RS-CVR, resting-state cerebrovascular reactivity; lrFCD, long-range functional connectivity density; L-SOG, left superior occipital gyrus; L-MOG, left middle occipital gyrus; L-IOG, left inferior occipital gyrus; R-IOG, right inferior occipital gyrus; MMSE, Mini Mental State Examination; AVLT, Auditory Verbal Learning Test; CFT, Rey-Osterrieth Complex Figure Test; CDT, Clock Drawing Test; AFT, Animal Fluency Test; BNT, 30-item Boston Naming Test; SDMT, Symbol Digit Modality Test; TMT-A, Trail Making Test A; TMT-B, Trail Making Test B; SCWT, Stroop Color-Word Test.


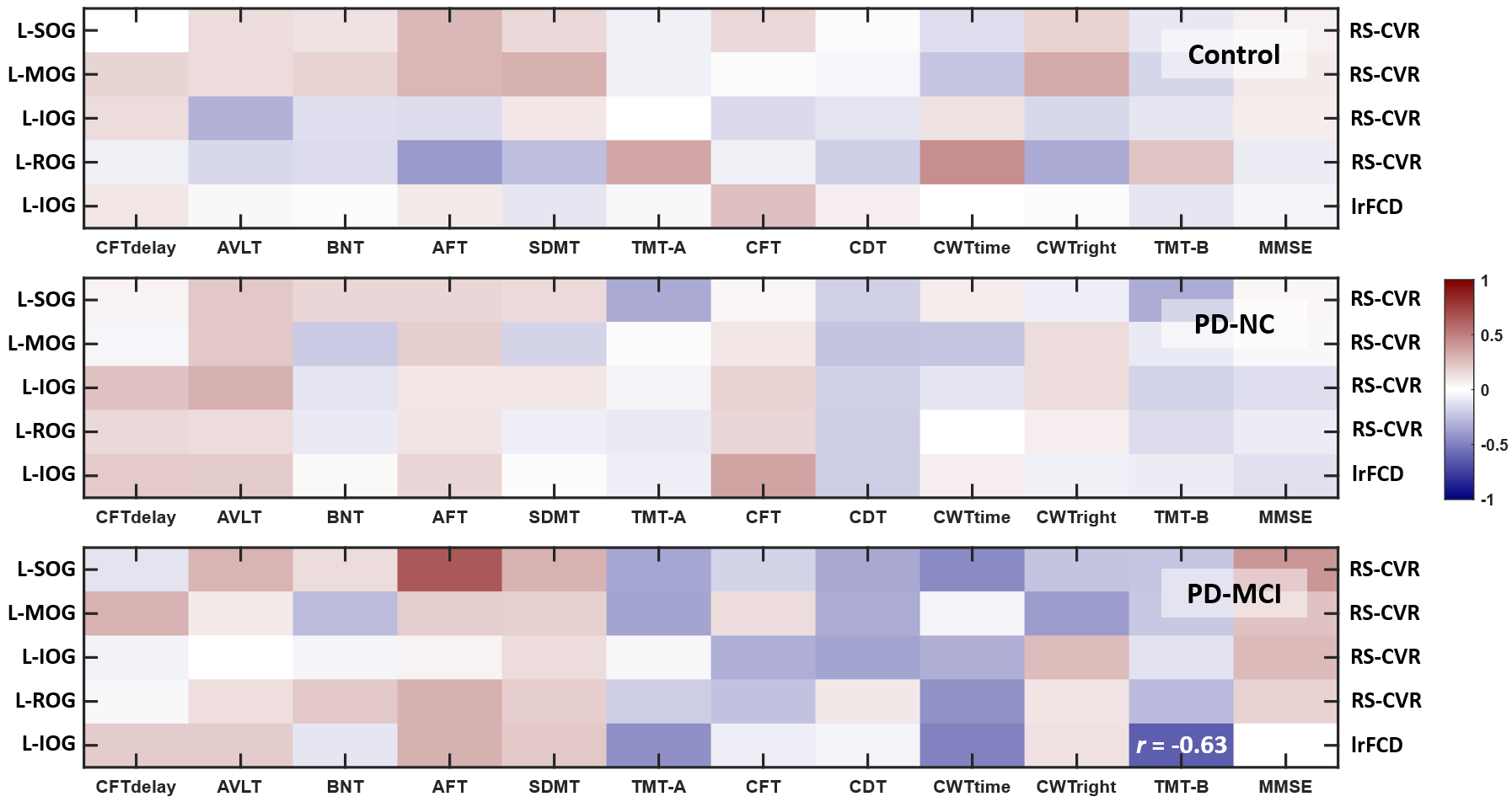


**FIG. S3.** Bivariate Spearman correlation analysis between imaging metrics extracted from the arterial territory template and 12 neuropsychological evaluations. CWT-time demonstrated significant negative correlations with RS-CVR in the distal territory of the RPCA (*r* = -0.7091, *p*FDR = 0.0304) and the middle territory of the LPCA (*r* = -0.6634, *p*FDR = 0.0440). A positive correlation was observed between ALFF in the proximal territory of the LPCA and TMT-A (*r* = 0.6538, *p*FDR = 0.0359). Abbreviations: RS-CVR, resting-state cerebrovascular reactivity; ALFF, amplitude of low-frequency fluctuations; RPCA, right posterior cerebral artery; LPCA, left posterior cerebral artery; p, proximal territory; m, middle territory; d, distal territory; MMSE, Mini Mental State Examination; AVLT, Auditory Verbal Learning Test; CFT, Rey-Osterrieth Complex Figure Test; CDT, Clock Drawing Test; AFT, Animal Fluency Test; BNT, 30-item Boston Naming Test; SDMT, Symbol Digit Modality Test; TMT-A, Trail Making Test A; TMT-B, Trail Making Test B; SCWT, Stroop Color-Word Test.


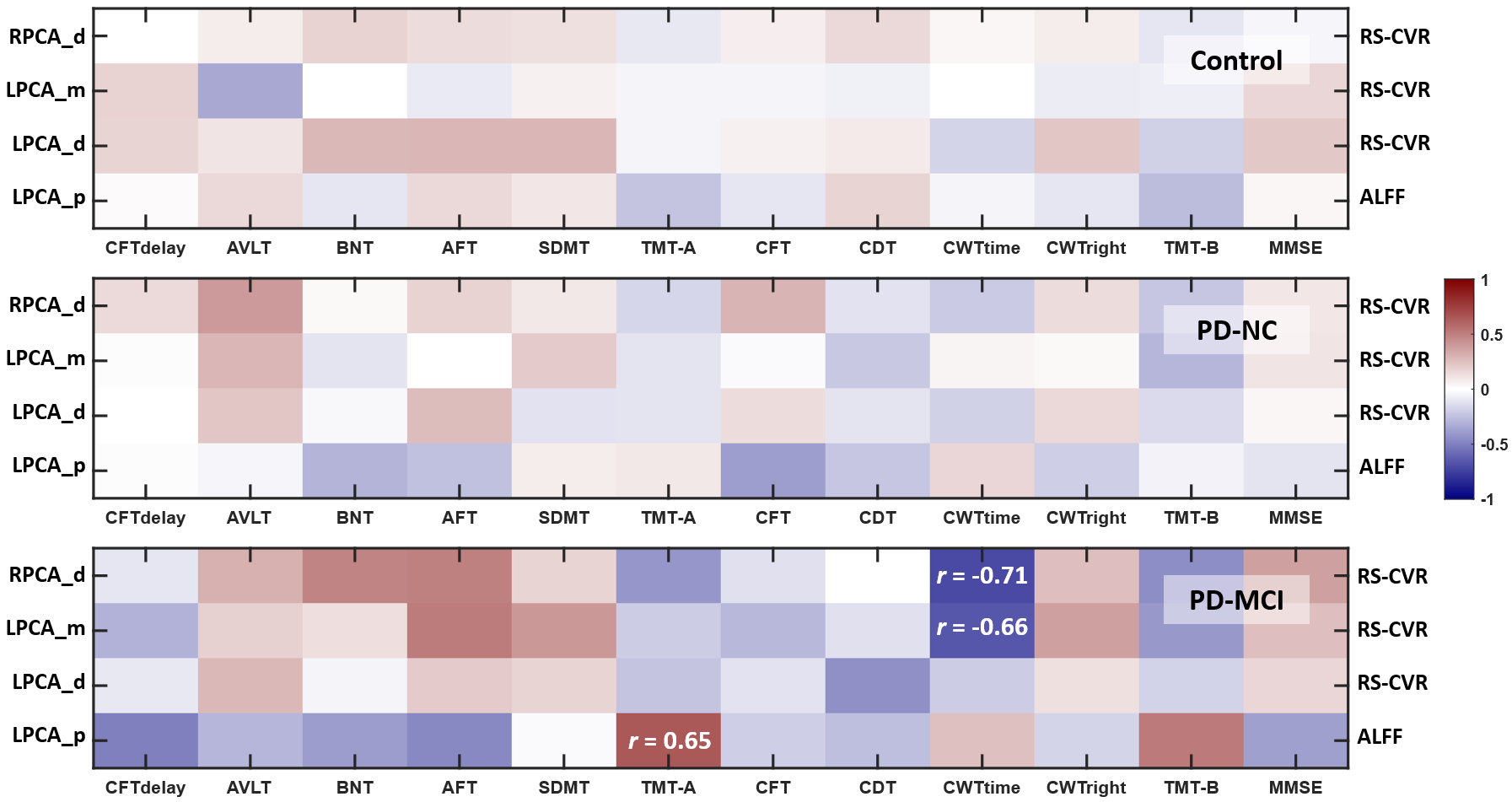


**FIG. S4.** ROC analysis. The three predictive indicators determined through LASSO regression were cerebrovascular reactivity (CVR) of the left inferior occipital gyrus (L-IOG), CVR of the middle territory of left posterior cerebral artery (PCA), and amplitude of low-frequency fluctuations (ALFF) of the proximal territory of left PCA. The L-IOG CVR demonstrated the best independent predictive performance and achieved an area under the curve (AUC) comparable to the combination of all three indicators.


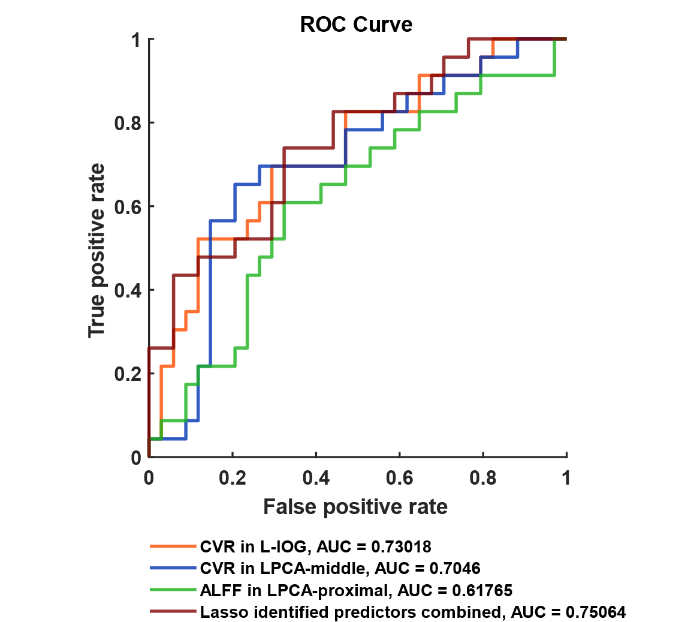


**TABLE S1.** Assessment of inter-rater reproducibility for arterial radius independently measured by two operators in PD-MCI (n = 25), along with the evaluation of measurement differences using the non-parametric Wilcoxon signed rank test.

| **Morphology Metrics** | **Intra-class coefficients of operators** | **Wilcoxon signed rank test *p* value** |
| --- | --- | --- |
| RICA radius | 0.84 (95%CI [0.67;0.93], *P* < 0.001 | 0.808 |
| LICA radius | 0.80 (95%CI [0.59;0.91], *P* < 0.001 | 0.563 |
| RACA-A1 radius | 0.89 (95%CI [0.75;0.95], *P* < 0.001 | 0.741 |
| LACA-A1 radius | 0.90 (95%CI [0.79;0.96], *P* < 0.001 | 0.338 |
| RMCA-M1 radius | 0.90 (95%CI [0.78;0.96], *P* < 0.001 | 0.412 |
| LMCA-M1 radius | 0.66 (95%CI [0.36;0.84], *P* < 0.001 | 0.274 |
| RPCA-P1 radius | 0.73 (95%CI [0.42;0.88], *P* < 0.001 | 0.184 |
| LPCA-P1 radius | 0.69 (95%CI [0.38;0.85], *P* < 0.001 | 0.614 |
| RPCA-P2 radius | 0.45 (95%CI [0.08;0.72], *P* = 0.010 | 0.999 |
| LPCA-P2 radius | 0.81 (95%CI [0.60;0.91], *P* < 0.001 | 0.189 |
| BA radius | 0.95 (95%CI [0.90;0.98], *P* < 0.001 | 0.238 |

**TABLE S2.** Comparison of resting-state cerebrovascular reactivity (CVR) maps among healthy controls (HCs), Parkinson's disease with normal cognition (PD-NC), and Parkinson's disease with mild cognitive impairment (PD-MCI) groups based on the AAL3 atlas. The non-parametric Kruskal-Wallis test was used for comparisons among the three groups, with FDR-corrected significant results indicated in bold. Region numbering and anatomical labels were aligned with the original AAL3 atlas without modification. Chi-sq: Chi-square; df: degree of freedom.

| Label in AAL3 | Chi-sq | df | p value | FDR Corrected p value |
| --- | --- | --- | --- | --- |
| 1 Precentral_L 1 | 1.5637 | 2 | 0.4576 | 0.8536 |
| 2 Precentral_R 2 | 0.9946 | 2 | 0.6082 | 0.9016 |
| 3 Frontal_Sup_2_L 3 | 0.6359 | 2 | 0.7276 | 0.9522 |
| 4 Frontal_Sup_2_R 4 | 0.6302 | 2 | 0.7297 | 0.9522 |
| 5 Frontal_Mid_2_L 5 | 1.5674 | 2 | 0.4567 | 0.8536 |
| 6 Frontal_Mid_2_R 6 | 2.2009 | 2 | 0.3327 | 0.7376 |
| 7 Frontal_Inf_Oper_L 7 | 0.2538 | 2 | 0.8808 | 0.9703 |
| 8 Frontal_Inf_Oper_R 8 | 1.8165 | 2 | 0.4032 | 0.8244 |
| 9 Frontal_Inf_Tri_L 9 | 0.0808 | 2 | 0.9604 | 0.9772 |
| 10 Frontal_Inf_Tri_R 10 | 2.9218 | 2 | 0.232 | 0.6372 |
| 11 Frontal_Inf_Orb_2_L 11 | 2.535 | 2 | 0.2815 | 0.7166 |
| 12 Frontal_Inf_Orb_2_R 12 | 3.9436 | 2 | 0.1392 | 0.5692 |
| 13 Rolandic_Oper_L 13 | 0.0742 | 2 | 0.9636 | 0.9772 |
| 14 Rolandic_Oper_R 14 | 0.1355 | 2 | 0.9345 | 0.9703 |
| 15 Supp_Motor_Area_L 15 | 0.2621 | 2 | 0.8772 | 0.9703 |
| 16 Supp_Motor_Area_R 16 | 1.8463 | 2 | 0.3973 | 0.8244 |
| 17 Olfactory_L 17 | 4.6682 | 2 | 0.0969 | 0.4953 |
| 18 Olfactory_R 18 | 0.7008 | 2 | 0.7044 | 0.9461 |
| 19 Frontal_Sup_Medial_L 19 | 5.0683 | 2 | 0.0793 | 0.4589 |
| 20 Frontal_Sup_Medial_R 20 | 3.9567 | 2 | 0.1383 | 0.5692 |
| 21 Frontal_Med_Orb_L 21 | 7.2979 | 2 | 0.026 | 0.2435 |
| 22 Frontal_Med_Orb_R 22 | 1.7587 | 2 | 0.415 | 0.8349 |
| 23 Rectus_L 23 | 1.2207 | 2 | 0.5432 | 0.8921 |
| 24 Rectus_R 24 | 2.4281 | 2 | 0.297 | 0.7179 |
| 25 OFCmed_L 25 | 3.2941 | 2 | 0.1926 | 0.6197 |
| 26 OFCmed_R 26 | 0.8788 | 2 | 0.6444 | 0.9192 |
| 27 OFCant_L 27 | 1.612 | 2 | 0.4466 | 0.8536 |
| 28 OFCant_R 28 | 1.0683 | 2 | 0.5862 | 0.8921 |
| 29 OFCpost_L 29 | 1.6875 | 2 | 0.4301 | 0.8419 |
| 30 OFCpost_R 30 | 0.3277 | 2 | 0.8489 | 0.9703 |
| 31 OFClat_L 31 | 1.4539 | 2 | 0.4834 | 0.875 |
| 32 OFClat_R 32 | 0.7888 | 2 | 0.6741 | 0.9396 |
| 33 Insula_L 33 | 0.2891 | 2 | 0.8654 | 0.9703 |
| 34 Insula_R 34 | 2.7034 | 2 | 0.2588 | 0.6803 |
| 37 Cingulate_Mid_L 37 | 0.8873 | 2 | 0.6417 | 0.9192 |
| 38 Cingulate_Mid_R 38 | 1.2882 | 2 | 0.5251 | 0.8921 |
| 39 Cingulate_Post_L 39 | 9.8755 | 2 | 0.0072 | 0.1164 |
| 40 Cingulate_Post_R 40 | 9.0664 | 2 | 0.0107 | 0.1353 |
| 41 Hippocampus_L 41 | 1.1249 | 2 | 0.5698 | 0.8921 |
| 42 Hippocampus_R 42 | 0.2262 | 2 | 0.8931 | 0.9703 |
| 43 ParaHippocampal_L 43 | 3.2884 | 2 | 0.1932 | 0.6197 |
| 44 ParaHippocampal_R 44 | 1.2263 | 2 | 0.5416 | 0.8921 |
| 45 Amygdala_L 45 | 3.0726 | 2 | 0.2152 | 0.6197 |
| 46 Amygdala_R 46 | 0.2238 | 2 | 0.8941 | 0.9703 |
| 47 Calcarine_L 47 | 4.2022 | 2 | 0.1223 | 0.5359 |
| 48 Calcarine_R 48 | 7.1309 | 2 | 0.0283 | 0.2435 |
| 49 Cuneus_L 49 | 4.9394 | 2 | 0.0846 | 0.4718 |
| 50 Cuneus_R 50 | 6.6387 | 2 | 0.0362 | 0.2774 |
| 51 Lingual_L 51 | 11.6922 | 2 | 0.0029 | 0.0662 |
| 52 Lingual_R 52 | 9.762 | 2 | 0.0076 | 0.1164 |
| 53 Occipital_Sup_L 53 | **16.0717** | **2** | **0.0003** | **0.0119** |
| 54 Occipital_Sup_R 54 | 7.2889 | 2 | 0.0261 | 0.2435 |
| 55 Occipital_Mid_L 55 | **16.2509** | **2** | **0.0003** | **0.0119** |
| 56 Occipital_Mid_R 56 | 5.8554 | 2 | 0.0535 | 0.3939 |
| 57 Occipital_Inf_L 57 | **19.5974** | **2** | **1.00E-04** | **0.0069** |
| 58 Occipital_Inf_R 58 | **15.4476** | **2** | **0.0004** | **0.0136** |
| 59 Fusiform_L 59 | 3.1063 | 2 | 0.2116 | 0.6197 |
| 60 Fusiform_R 60 | 5.3449 | 2 | 0.0691 | 0.454 |
| 61 Postcentral_L 61 | 1.0667 | 2 | 0.5866 | 0.8921 |
| 62 Postcentral_R 62 | 3.0772 | 2 | 0.2147 | 0.6197 |
| 63 Parietal_Sup_L 63 | 1.2237 | 2 | 0.5423 | 0.8921 |
| 64 Parietal_Sup_R 64 | 2.7957 | 2 | 0.2471 | 0.6668 |
| 65 Parietal_Inf_L 65 | 0.7962 | 2 | 0.6716 | 0.9396 |
| 66 Parietal_Inf_R 66 | 1.1739 | 2 | 0.556 | 0.8921 |
| 67 SupraMarginal_L 67 | 1.0393 | 2 | 0.5947 | 0.897 |
| 68 SupraMarginal_R 68 | 0.1607 | 2 | 0.9228 | 0.9703 |
| 69 Angular_L 69 | 0.1266 | 2 | 0.9386 | 0.9703 |
| 70 Angular_R 70 | 4.4089 | 2 | 0.1103 | 0.5204 |
| 71 Precuneus_L 71 | 2.7722 | 2 | 0.25 | 0.6668 |
| 72 Precuneus_R 72 | 3.7791 | 2 | 0.1511 | 0.5917 |
| 73 Paracentral_Lobule_L 73 | 0.7022 | 2 | 0.7039 | 0.9461 |
| 74 Paracentral_Lobule_R 74 | 4.7489 | 2 | 0.0931 | 0.4944 |
| 75 Caudate_L 75 | 0.1804 | 2 | 0.9138 | 0.9703 |
| 76 Caudate_R 76 | 0.0424 | 2 | 0.979 | 0.979 |
| 77 Putamen_L 77 | 11.4654 | 2 | 0.0032 | 0.0662 |
| 78 Putamen_R 78 | 7.0731 | 2 | 0.0291 | 0.2435 |
| 79 Pallidum_L 79 | 8.0718 | 2 | 0.0177 | 0.2032 |
| 80 Pallidum_R 80 | 9.445 | 2 | 0.0089 | 0.1259 |
| 83 Heschl_L 83 | 3.2552 | 2 | 0.1964 | 0.6197 |
| 84 Heschl_R 84 | 0.7668 | 2 | 0.6815 | 0.9409 |
| 85 Temporal_Sup_L 85 | 4.5897 | 2 | 0.1008 | 0.5012 |
| 86 Temporal_Sup_R 86 | 2.3792 | 2 | 0.3043 | 0.7179 |
| 87 Temporal_Pole_Sup_L 87 | 1.2501 | 2 | 0.5352 | 0.8921 |
| 88 Temporal_Pole_Sup_R 88 | 2.9693 | 2 | 0.2266 | 0.6317 |
| 89 Temporal_Mid_L 89 | 5.4612 | 2 | 0.0652 | 0.454 |
| 90 Temporal_Mid_R 90 | 3.6772 | 2 | 0.159 | 0.6097 |
| 91 Temporal_Pole_Mid_L 91 | 1.9193 | 2 | 0.383 | 0.8195 |
| 92 Temporal_Pole_Mid_R 92 | 1.2359 | 2 | 0.539 | 0.8921 |
| 93 Temporal_Inf_L 93 | 0.924 | 2 | 0.63 | 0.9128 |
| 94 Temporal_Inf_R 94 | 2.1738 | 2 | 0.3373 | 0.7388 |
| 95 Cerebellum_Crus1_L 95 | 1.5561 | 2 | 0.4593 | 0.8536 |
| 96 Cerebellum_Crus1_R 96 | 0.8444 | 2 | 0.6556 | 0.9279 |
| 97 Cerebellum_Crus2_L 97 | 4.4854 | 2 | 0.1062 | 0.5141 |
| 98 Cerebellum_Crus2_R 98 | 5.3499 | 2 | 0.0689 | 0.454 |
| 99 Cerebellum_3_L 99 | 3.129 | 2 | 0.2092 | 0.6197 |
| 100 Cerebellum_3_R 100 | 1.4469 | 2 | 0.4851 | 0.875 |
| 101 Cerebellum_4_5_L 101 | 0.0552 | 2 | 0.9728 | 0.9781 |
| 102 Cerebellum_4_5_R 102 | 0.0999 | 2 | 0.9513 | 0.9772 |
| 103 Cerebellum_6_L 103 | 2.302 | 2 | 0.3163 | 0.7275 |
| 104 Cerebellum_6_R 104 | 0.729 | 2 | 0.6946 | 0.9461 |
| 105 Cerebellum_7b_L 105 | 0.2991 | 2 | 0.8611 | 0.9703 |
| 106 Cerebellum_7b_R 106 | 1.0777 | 2 | 0.5834 | 0.8921 |
| 107 Cerebellum_8_L 107 | 0.4838 | 2 | 0.7851 | 0.9567 |
| 108 Cerebellum_8_R 108 | 1.4812 | 2 | 0.4768 | 0.875 |
| 109 Cerebellum_9_L 109 | 7.0733 | 2 | 0.0291 | 0.2435 |
| 110 Cerebellum_9_R 110 | 4.1374 | 2 | 0.1264 | 0.5407 |
| 111 Cerebellum_10_L 111 | 9.0144 | 2 | 0.011 | 0.1353 |
| 112 Cerebellum_10_R 112 | 1.2785 | 2 | 0.5277 | 0.8921 |
| 113 Vermis_1_2 113 | 1.7301 | 2 | 0.421 | 0.8349 |
| 114 Vermis_3 114 | 0.2435 | 2 | 0.8854 | 0.9703 |
| 115 Vermis_4_5 115 | 2.4614 | 2 | 0.2921 | 0.7166 |
| 116 Vermis_6 116 | 3.4407 | 2 | 0.179 | 0.6197 |
| 117 Vermis_7 117 | 0.2031 | 2 | 0.9034 | 0.9703 |
| 118 Vermis_8 118 | 3.153 | 2 | 0.2067 | 0.6197 |
| 119 Vermis_9 119 | 5.1314 | 2 | 0.0769 | 0.4589 |
| 120 Vermis_10 120 | 0.15 | 2 | 0.9277 | 0.9703 |
| 121 Thal_AV_L 121 | 0.9995 | 2 | 0.6067 | 0.9016 |
| 122 Thal_AV_R 122 | 0.6629 | 2 | 0.7179 | 0.9503 |
| 123 Thal_LP_L 123 | 0.5608 | 2 | 0.7555 | 0.9535 |
| 124 Thal_LP_R 124 | 3.806 | 2 | 0.1491 | 0.5917 |
| 125 Thal_VA_L 125 | 2.2398 | 2 | 0.3263 | 0.7322 |
| 126 Thal_VA_R 126 | 0.9663 | 2 | 0.6168 | 0.9016 |
| 127 Thal_VL_L 127 | 0.756 | 2 | 0.6852 | 0.9409 |
| 128 Thal_VL_R 128 | 5.1777 | 2 | 0.0751 | 0.4589 |
| 129 Thal_VPL_L 129 | 1.3579 | 2 | 0.5071 | 0.8921 |
| 130 Thal_VPL_R 130 | 0.1416 | 2 | 0.9316 | 0.9703 |
| 131 Thal_IL_L 131 | 1.8176 | 2 | 0.403 | 0.8244 |
| 132 Thal_IL_R 132 | 1.1972 | 2 | 0.5496 | 0.8921 |
| 133 Thal_Re_L 133 | 2.4674 | 2 | 0.2912 | 0.7166 |
| 134 Thal_Re_R 134 | 2.571 | 2 | 0.2765 | 0.7166 |
| 135 Thal_MDm_L 135 | 2.3112 | 2 | 0.3149 | 0.7275 |
| 136 Thal_MDm_R 136 | 3.0985 | 2 | 0.2124 | 0.6197 |
| 137 Thal_MDl_L 137 | 2.4945 | 2 | 0.2873 | 0.7166 |
| 138 Thal_MDl_R 138 | 9.8744 | 2 | 0.0072 | 0.1164 |
| 139 Thal_LGN_L 139 | 3.0526 | 2 | 0.2173 | 0.6197 |
| 140 Thal_LGN_R 140 | 0.5039 | 2 | 0.7773 | 0.9535 |
| 141 Thal_MGN_L 141 | 0.5465 | 2 | 0.7609 | 0.9535 |
| 142 Thal_MGN_R 142 | 0.5905 | 2 | 0.7443 | 0.9535 |
| 143 Thal_PuI_L 143 | 0.068 | 2 | 0.9666 | 0.9772 |
| 144 Thal_PuI_R 144 | 0.3959 | 2 | 0.8204 | 0.9703 |
| 145 Thal_PuM_L 145 | 0.1709 | 2 | 0.9181 | 0.9703 |
| 146 Thal_PuM_R 146 | 1.1062 | 2 | 0.5752 | 0.8921 |
| 147 Thal_PuA_L 147 | 0.2268 | 2 | 0.8928 | 0.9703 |
| 148 Thal_PuA_R 148 | 1.2005 | 2 | 0.5487 | 0.8921 |
| 149 Thal_PuL_L 149 | 0.5132 | 2 | 0.7737 | 0.9535 |
| 150 Thal_PuL_R 150 | 0.3487 | 2 | 0.84 | 0.9703 |
| 151 ACC_sub_L 151 | 0.1844 | 2 | 0.9119 | 0.9703 |
| 152 ACC_sub_R 152 | 2.3826 | 2 | 0.3038 | 0.7179 |
| 153 ACC_pre_L 153 | 0.3866 | 2 | 0.8242 | 0.9703 |
| 154 ACC_pre_R 154 | 1.1594 | 2 | 0.5601 | 0.8921 |
| 155 ACC_sup_L 155 | 0.1891 | 2 | 0.9098 | 0.9703 |
| 156 ACC_sup_R 156 | 0.2951 | 2 | 0.8628 | 0.9703 |
| 157 N_Acc_L 157 | 4.3569 | 2 | 0.1132 | 0.5208 |
| 158 N_Acc_R 158 | 0.5197 | 2 | 0.7712 | 0.9535 |
| 159 VTA_L 159 | 0.5984 | 2 | 0.7414 | 0.9535 |
| 160 VTA_R 160 | 0.6679 | 2 | 0.7161 | 0.9503 |
| 161 SN_pc_L 161 | 1.5764 | 2 | 0.4547 | 0.8536 |
| 162 SN_pc_R 162 | 0.2224 | 2 | 0.8947 | 0.9703 |
| 163 SN_pr_L 163 | 0.295 | 2 | 0.8629 | 0.9703 |
| 164 SN_pr_R 164 | 0.3166 | 2 | 0.8536 | 0.9703 |
| 165 Red_N_L 165 | 3.2174 | 2 | 0.2001 | 0.6197 |
| 166 Red_N_R 166 | 2.25 | 2 | 0.3247 | 0.7322 |
| 167 LC_L 167 | 0.5773 | 2 | 0.7493 | 0.9535 |
| 168 LC_R 168 | 0.9646 | 2 | 0.6174 | 0.9016 |
| 169 Raphe_D 169 | 2.0665 | 2 | 0.3558 | 0.7703 |
| 170 Raphe_M 170 | 0.2922 | 2 | 0.8641 | 0.9703 |

**TABLE S3.** Comparison of resting-state cerebrovascular reactivity (CVR) maps among healthy controls (HCs), Parkinson's disease with normal cognition (PD-NC), and Parkinson's disease with mild cognitive impairment (PD-MCI) groups based on the arterial territory template. The non-parametric Kruskal-Wallis test was used for comparisons among the three groups, with FDR-corrected significant results indicated in bold. Chi-sq: Chi-square; df: degree of freedom.

| Arterial Territories | Chi-sq | df | p value | FDR Corrected p value |
| --- | --- | --- | --- | --- |
| RACA_p | 3.4391 | 2 | 0.1791 | 0.6197 |
| RACA_m | 1.8207 | 2 | 0.4024 | 0.8244 |
| RACA_d | 7.4293 | 2 | 0.0244 | 0.2435 |
| RMCA_p | 3.251 | 2 | 0.1968 | 0.6197 |
| RMCA_m | 3.1214 | 2 | 0.21 | 0.6197 |
| RMCA_d | 1.0748 | 2 | 0.5843 | 0.8921 |
| RPCA_p | 3.038 | 2 | 0.2189 | 0.6197 |
| RPCA_m | 6.8961 | 2 | 0.0318 | 0.2545 |
| RPCA_d | **17.6382** | **2** | **0.0001** | **0.0091** |
| LACA_p | 5.056 | 2 | 0.0798 | 0.4589 |
| LACA_m | 1.1715 | 2 | 0.5567 | 0.8921 |
| LACA_d | 4.728 | 2 | 0.094 | 0.4944 |
| LMCA_p | 3.4374 | 2 | 0.1793 | 0.6197 |
| LMCA_m | 1.7255 | 2 | 0.422 | 0.8349 |
| LMCA_d | 0.5394 | 2 | 0.7636 | 0.9535 |
| LPCA_p | 4.2962 | 2 | 0.1167 | 0.5238 |
| LPCA_m | **13.6963** | **2** | **0.0011** | **0.0279** |
| LPCA_d | **18.9825** | **2** | **0.0001** | **0.0069** |

Abbreviations: R, right; L, left; p, proximal territory; m, middle territory; d, distal territory; MCA, middle cerebral arteries; ACA, anterior cerebral arteries; PCA, posterior cerebral arteries.

**TABLE S4.** Comparison of cerebral blood flow (CBF) maps among healthy controls (HCs), Parkinson's disease with normal cognition (PD-NC), and Parkinson's disease with mild cognitive impairment (PD-MCI) groups based on the AAL3 atlas. The non-parametric Kruskal-Wallis test was used for comparisons among the three groups, with FDR-corrected significant results indicated in bold. Region numbering and anatomical labels were aligned with the original AAL3 atlas without modification. Chi-sq: Chi-square; df: degree of freedom.

| Label in AAL3 | Chi-sq | df | p value | FDR Corrected p value |
| --- | --- | --- | --- | --- |
| 1 Precentral_L 1 | 2.7265 | 2 | 0.2558 | 0.3923 |
| 2 Precentral_R 2 | 2.7437 | 2 | 0.2536 | 0.3922 |
| 3 Frontal_Sup_2_L 3 | 3.9183 | 2 | 0.141 | 0.3304 |
| 4 Frontal_Sup_2_R 4 | 3.9221 | 2 | 0.1407 | 0.3304 |
| 5 Frontal_Mid_2_L 5 | 4.5993 | 2 | 0.1003 | 0.3217 |
| 6 Frontal_Mid_2_R 6 | 3.3281 | 2 | 0.1894 | 0.344 |
| 7 Frontal_Inf_Oper_L 7 | 5.9237 | 2 | 0.0517 | 0.3217 |
| 8 Frontal_Inf_Oper_R 8 | 3.8067 | 2 | 0.1491 | 0.3304 |
| 9 Frontal_Inf_Tri_L 9 | 6.7569 | 2 | 0.0341 | 0.3005 |
| 10 Frontal_Inf_Tri_R 10 | 3.9836 | 2 | 0.1364 | 0.3304 |
| 11 Frontal_Inf_Orb_2_L 11 | 11.3972 | 2 | 0.0034 | 0.2055 |
| 12 Frontal_Inf_Orb_2_R 12 | 7.2145 | 2 | 0.0271 | 0.3005 |
| 13 Rolandic_Oper_L 13 | 3.0197 | 2 | 0.2209 | 0.3594 |
| 14 Rolandic_Oper_R 14 | 2.1894 | 2 | 0.3346 | 0.4561 |
| 15 Supp_Motor_Area_L 15 | 1.8052 | 2 | 0.4055 | 0.5368 |
| 16 Supp_Motor_Area_R 16 | 0.8653 | 2 | 0.6488 | 0.7415 |
| 17 Olfactory_L 17 | 3.0484 | 2 | 0.2178 | 0.3586 |
| 18 Olfactory_R 18 | 3.3104 | 2 | 0.1911 | 0.344 |
| 19 Frontal_Sup_Medial_L 19 | 4.1871 | 2 | 0.1232 | 0.3304 |
| 20 Frontal_Sup_Medial_R 20 | 1.3925 | 2 | 0.4985 | 0.6155 |
| 21 Frontal_Med_Orb_L 21 | 5.1828 | 2 | 0.0749 | 0.3217 |
| 22 Frontal_Med_Orb_R 22 | 3.166 | 2 | 0.2054 | 0.3565 |
| 23 Rectus_L 23 | 3.7834 | 2 | 0.1508 | 0.3304 |
| 24 Rectus_R 24 | 3.1168 | 2 | 0.2105 | 0.3586 |
| 25 OFCmed_L 25 | 0.4999 | 2 | 0.7788 | 0.848 |
| 26 OFCmed_R 26 | 2.3435 | 2 | 0.3098 | 0.4385 |
| 27 OFCant_L 27 | 0.5584 | 2 | 0.7564 | 0.8334 |
| 28 OFCant_R 28 | 1.1125 | 2 | 0.5734 | 0.6806 |
| 29 OFCpost_L 29 | 5.7803 | 2 | 0.0556 | 0.3217 |
| 30 OFCpost_R 30 | 6.8077 | 2 | 0.0332 | 0.3005 |
| 31 OFClat_L 31 | 8.0014 | 2 | 0.0183 | 0.3005 |
| 32 OFClat_R 32 | 7.8431 | 2 | 0.0198 | 0.3005 |
| 33 Insula_L 33 | 7.0514 | 2 | 0.0294 | 0.3005 |
| 34 Insula_R 34 | 6.3667 | 2 | 0.0414 | 0.3005 |
| 37 Cingulate_Mid_L 37 | 3.0439 | 2 | 0.2183 | 0.3586 |
| 38 Cingulate_Mid_R 38 | 2.0319 | 2 | 0.3621 | 0.4898 |
| 39 Cingulate_Post_L 39 | 5.0213 | 2 | 0.0812 | 0.3217 |
| 40 Cingulate_Post_R 40 | 4.1998 | 2 | 0.1225 | 0.3304 |
| 41 Hippocampus_L 41 | 2.3813 | 2 | 0.304 | 0.4347 |
| 42 Hippocampus_R 42 | 2.2886 | 2 | 0.3184 | 0.4439 |
| 43 ParaHippocampal_L 43 | 4.474 | 2 | 0.1068 | 0.3217 |
| 44 ParaHippocampal_R 44 | 3.8651 | 2 | 0.1448 | 0.3304 |
| 45 Amygdala_L 45 | 1.2955 | 2 | 0.5232 | 0.6366 |
| 46 Amygdala_R 46 | 0.5073 | 2 | 0.7759 | 0.848 |
| 47 Calcarine_L 47 | 6.3181 | 2 | 0.0425 | 0.3005 |
| 48 Calcarine_R 48 | 5.6926 | 2 | 0.0581 | 0.3217 |
| 49 Cuneus_L 49 | 4.6564 | 2 | 0.0975 | 0.3217 |
| 50 Cuneus_R 50 | 5.0451 | 2 | 0.0803 | 0.3217 |
| 51 Lingual_L 51 | 4.9903 | 2 | 0.0825 | 0.3217 |
| 52 Lingual_R 52 | 5.1162 | 2 | 0.0775 | 0.3217 |
| 53 Occipital_Sup_L 53 | 4.9792 | 2 | 0.0829 | 0.3217 |
| 54 Occipital_Sup_R 54 | 6.5138 | 2 | 0.0385 | 0.3005 |
| 55 Occipital_Mid_L 55 | 3.5964 | 2 | 0.1656 | 0.3369 |
| 56 Occipital_Mid_R 56 | 2.6751 | 2 | 0.2625 | 0.3959 |
| 57 Occipital_Inf_L 57 | 7.5512 | 2 | 2.29E-02 | 0.3005 |
| 58 Occipital_Inf_R 58 | 4.5878 | 2 | 0.1009 | 0.3217 |
| 59 Fusiform_L 59 | 3.8351 | 2 | 0.147 | 0.3304 |
| 60 Fusiform_R 60 | 3.0554 | 2 | 0.217 | 0.3586 |
| 61 Postcentral_L 61 | 4.0395 | 2 | 0.1327 | 0.3304 |
| 62 Postcentral_R 62 | 2.8011 | 2 | 0.2465 | 0.3843 |
| 63 Parietal_Sup_L 63 | 3.6078 | 2 | 0.1647 | 0.3369 |
| 64 Parietal_Sup_R 64 | 3.8462 | 2 | 0.1462 | 0.3304 |
| 65 Parietal_Inf_L 65 | 2.4866 | 2 | 0.2884 | 0.4245 |
| 66 Parietal_Inf_R 66 | 3.4384 | 2 | 0.1792 | 0.3438 |
| 67 SupraMarginal_L 67 | 4.4706 | 2 | 0.107 | 0.3217 |
| 68 SupraMarginal_R 68 | 4.4009 | 2 | 0.1108 | 0.3217 |
| 69 Angular_L 69 | 3.709 | 2 | 0.1565 | 0.3369 |
| 70 Angular_R 70 | 3.2826 | 2 | 0.1937 | 0.344 |
| 71 Precuneus_L 71 | 3.8229 | 2 | 0.1479 | 0.3304 |
| 72 Precuneus_R 72 | 3.5839 | 2 | 0.1666 | 0.3369 |
| 73 Paracentral_Lobule_L 73 | 5.0057 | 2 | 0.0819 | 0.3217 |
| 74 Paracentral_Lobule_R 74 | 1.9361 | 2 | 0.3798 | 0.5101 |
| 75 Caudate_L 75 | 5.3582 | 2 | 0.0686 | 0.3217 |
| 76 Caudate_R 76 | 3.4884 | 2 | 0.1748 | 0.3438 |
| 77 Putamen_L 77 | 0.0333 | 2 | 0.9835 | 0.9889 |
| 78 Putamen_R 78 | 0.0886 | 2 | 0.9567 | 0.9771 |
| 79 Pallidum_L 79 | 1.4464 | 2 | 0.4852 | 0.6136 |
| 80 Pallidum_R 80 | 0.1905 | 2 | 0.9091 | 0.9505 |
| 83 Heschl_L 83 | 12.4856 | 2 | 0.0019 | 0.1789 |
| 84 Heschl_R 84 | 4.5156 | 2 | 0.1046 | 0.3217 |
| 85 Temporal_Sup_L 85 | 7.197 | 2 | 0.0274 | 0.3005 |
| 86 Temporal_Sup_R 86 | 4.1609 | 2 | 0.1249 | 0.3304 |
| 87 Temporal_Pole_Sup_L 87 | 14.0633 | 2 | 0.0009 | 0.1626 |
| 88 Temporal_Pole_Sup_R 88 | 8.7518 | 2 | 0.0126 | 0.3005 |
| 89 Temporal_Mid_L 89 | 6.0235 | 2 | 0.0492 | 0.3217 |
| 90 Temporal_Mid_R 90 | 5.4323 | 2 | 0.0661 | 0.3217 |
| 91 Temporal_Pole_Mid_L 91 | 9.3349 | 2 | 0.0094 | 0.3005 |
| 92 Temporal_Pole_Mid_R 92 | 6.5224 | 2 | 0.0383 | 0.3005 |
| 93 Temporal_Inf_L 93 | 5.8317 | 2 | 0.0542 | 0.3217 |
| 94 Temporal_Inf_R 94 | 6.381 | 2 | 0.0412 | 0.3005 |
| 95 Cerebellum_Crus1_L 95 | 4.4363 | 2 | 0.1088 | 0.3217 |
| 96 Cerebellum_Crus1_R 96 | 3.9529 | 2 | 0.1386 | 0.3304 |
| 97 Cerebellum_Crus2_L 97 | 4.7597 | 2 | 0.0926 | 0.3217 |
| 98 Cerebellum_Crus2_R 98 | 3.477 | 2 | 0.1758 | 0.3438 |
| 99 Cerebellum_3_L 99 | 7.4206 | 2 | 0.0245 | 0.3005 |
| 100 Cerebellum_3_R 100 | 8.0031 | 2 | 0.0183 | 0.3005 |
| 101 Cerebellum_4_5_L 101 | 5.898 | 2 | 0.0524 | 0.3217 |
| 102 Cerebellum_4_5_R 102 | 4.8267 | 2 | 0.0895 | 0.3217 |
| 103 Cerebellum_6_L 103 | 4.4594 | 2 | 0.1076 | 0.3217 |
| 104 Cerebellum_6_R 104 | 4.3802 | 2 | 0.1119 | 0.3217 |
| 105 Cerebellum_7b_L 105 | 6.5545 | 2 | 0.0377 | 0.3005 |
| 106 Cerebellum_7b_R 106 | 3.2457 | 2 | 0.1973 | 0.3458 |
| 107 Cerebellum_8_L 107 | 4.4349 | 2 | 0.1089 | 0.3217 |
| 108 Cerebellum_8_R 108 | 4.343 | 2 | 0.114 | 0.3227 |
| 109 Cerebellum_9_L 109 | 1.8799 | 2 | 0.3907 | 0.5209 |
| 110 Cerebellum_9_R 110 | 1.4984 | 2 | 0.4728 | 0.6083 |
| 111 Cerebellum_10_L 111 | 6.3809 | 2 | 0.0412 | 0.3005 |
| 112 Cerebellum_10_R 112 | 4.7516 | 2 | 0.0929 | 0.3217 |
| 113 Vermis_1_2 113 | 2.8214 | 2 | 0.244 | 0.3837 |
| 114 Vermis_3 114 | 2.3764 | 2 | 0.3048 | 0.4347 |
| 115 Vermis_4_5 115 | 3.0042 | 2 | 0.2227 | 0.3594 |
| 116 Vermis_6 116 | 4.4572 | 2 | 0.1077 | 0.3217 |
| 117 Vermis_7 117 | 1.7525 | 2 | 0.4163 | 0.5433 |
| 118 Vermis_8 118 | 2.4699 | 2 | 0.2908 | 0.4245 |
| 119 Vermis_9 119 | 0.667 | 2 | 0.7164 | 0.7941 |
| 120 Vermis_10 120 | 0.9944 | 2 | 0.6082 | 0.7039 |
| 121 Thal_AV_L 121 | 2.5715 | 2 | 0.2764 | 0.4103 |
| 122 Thal_AV_R 122 | 1.3981 | 2 | 0.4971 | 0.6155 |
| 123 Thal_LP_L 123 | 3.6846 | 2 | 0.1585 | 0.3369 |
| 124 Thal_LP_R 124 | 0.9072 | 2 | 0.6353 | 0.7306 |
| 125 Thal_VA_L 125 | 3.6345 | 2 | 0.1625 | 0.3369 |
| 126 Thal_VA_R 126 | 1.4444 | 2 | 0.4857 | 0.6136 |
| 127 Thal_VL_L 127 | 3.4156 | 2 | 0.1813 | 0.3438 |
| 128 Thal_VL_R 128 | 1.2022 | 2 | 0.5482 | 0.6593 |
| 129 Thal_VPL_L 129 | 3.8257 | 2 | 0.1477 | 0.3304 |
| 130 Thal_VPL_R 130 | 1.0642 | 2 | 0.5874 | 0.6928 |
| 131 Thal_IL_L 131 | 0.3235 | 2 | 0.8507 | 0.8944 |
| 132 Thal_IL_R 132 | 0.781 | 2 | 0.6767 | 0.7669 |
| 133 Thal_Re_L 133 | 1.2855 | 2 | 0.5259 | 0.6366 |
| 134 Thal_Re_R 134 | 2.3187 | 2 | 0.3137 | 0.4406 |
| 135 Thal_MDm_L 135 | 1.6264 | 2 | 0.4434 | 0.5746 |
| 136 Thal_MDm_R 136 | 5.6482 | 2 | 0.0594 | 0.3217 |
| 137 Thal_MDl_L 137 | 0.3809 | 2 | 0.8266 | 0.8741 |
| 138 Thal_MDl_R 138 | 0.7315 | 2 | 0.6937 | 0.7783 |
| 139 Thal_LGN_L 139 | 0.7732 | 2 | 0.6793 | 0.7669 |
| 140 Thal_LGN_R 140 | 0.1583 | 2 | 0.9239 | 0.9551 |
| 141 Thal_MGN_L 141 | 1.1226 | 2 | 0.5705 | 0.6806 |
| 142 Thal_MGN_R 142 | 2.2516 | 2 | 0.3244 | 0.4488 |
| 143 Thal_PuI_L 143 | 0.0027 | 2 | 0.9987 | 0.9987 |
| 144 Thal_PuI_R 144 | 1.0273 | 2 | 0.5983 | 0.6968 |
| 145 Thal_PuM_L 145 | 0.4147 | 2 | 0.8127 | 0.8694 |
| 146 Thal_PuM_R 146 | 0.1686 | 2 | 0.9192 | 0.9551 |
| 147 Thal_PuA_L 147 | 0.6816 | 2 | 0.7112 | 0.7931 |
| 148 Thal_PuA_R 148 | 0.0495 | 2 | 0.9756 | 0.9863 |
| 149 Thal_PuL_L 149 | 1.3103 | 2 | 0.5194 | 0.6366 |
| 150 Thal_PuL_R 150 | 0.4511 | 2 | 0.7981 | 0.8638 |
| 151 ACC_sub_L 151 | 1.4395 | 2 | 0.4869 | 0.6136 |
| 152 ACC_sub_R 152 | 2.5711 | 2 | 0.2765 | 0.4103 |
| 153 ACC_pre_L 153 | 7.2028 | 2 | 0.0273 | 0.3005 |
| 154 ACC_pre_R 154 | 2.9228 | 2 | 0.2319 | 0.3711 |
| 155 ACC_sup_L 155 | 6.724 | 2 | 0.0347 | 0.3005 |
| 156 ACC_sup_R 156 | 2.4551 | 2 | 0.293 | 0.4245 |
| 157 N_Acc_L 157 | 3.3584 | 2 | 0.1865 | 0.344 |
| 158 N_Acc_R 158 | 2.2255 | 2 | 0.3287 | 0.4513 |
| 159 VTA_L 159 | 3.3314 | 2 | 0.1891 | 0.344 |
| 160 VTA_R 160 | 8.0168 | 2 | 0.0182 | 0.3005 |
| 161 SN_pc_L 161 | 0.0792 | 2 | 0.9612 | 0.9771 |
| 162 SN_pc_R 162 | 2.8928 | 2 | 0.2354 | 0.3734 |
| 163 SN_pr_L 163 | 0.1079 | 2 | 0.9475 | 0.974 |
| 164 SN_pr_R 164 | 1.4189 | 2 | 0.4919 | 0.6155 |
| 165 Red_N_L 165 | 2.675 | 2 | 0.2625 | 0.3959 |
| 166 Red_N_R 166 | 3.8843 | 2 | 0.1434 | 0.3304 |
| 167 LC_L 167 | 0.4012 | 2 | 0.8182 | 0.8703 |
| 168 LC_R 168 | 0.4317 | 2 | 0.8058 | 0.8671 |
| 169 Raphe_D 169 | 1.0319 | 2 | 0.5969 | 0.6968 |
| 170 Raphe_M 170 | 1.7538 | 2 | 0.4161 | 0.5433 |

**TABLE S5.** Comparison of cerebral blood flow (CBF) maps among healthy controls (HCs), Parkinson's disease with normal cognition (PD-NC), and Parkinson's disease with mild cognitive impairment (PD-MCI) groups based on the arterial territory template. The non-parametric Kruskal-Wallis test was used for comparisons among the three groups, with FDR-corrected significant results indicated in bold. Chi-sq: Chi-square; df: degree of freedom.

| Arterial Territories | Chi-sq | df | p value | FDR Corrected p value |
| --- | --- | --- | --- | --- |
| RACA_p | 3.0567 | 2 | 0.2169 | 0.3586 |
| RACA_m | 3.3126 | 2 | 0.1908 | 0.344 |
| RACA_d | 3.4263 | 2 | 0.1803 | 0.3438 |
| RMCA_p | 5.3102 | 2 | 0.0703 | 0.3217 |
| RMCA_m | 3.9066 | 2 | 0.1418 | 0.3304 |
| RMCA_d | 3.6363 | 2 | 0.1623 | 0.3369 |
| RPCA_p | 5.6347 | 2 | 0.0598 | 0.3217 |
| RPCA_m | 4.6926 | 2 | 0.0957 | 0.3217 |
| RPCA_d | 4.8781 | 2 | 0.0872 | 0.3217 |
| LACA_p | 3.5345 | 2 | 0.1708 | 0.3416 |
| LACA_m | 4.028 | 2 | 0.1335 | 0.3304 |
| LACA_d | 3.8323 | 2 | 0.1472 | 0.3304 |
| LMCA_p | 7.2827 | 2 | 0.0262 | 0.3005 |
| LMCA_m | 5.4731 | 2 | 0.0648 | 0.3217 |
| LMCA_d | 3.2751 | 2 | 0.1945 | 0.344 |
| LPCA_p | 5.3624 | 2 | 0.0685 | 0.3217 |
| LPCA_m | 4.7652 | 2 | 0.0923 | 0.3217 |
| LPCA_d | 3.0536 | 2 | 0.2172 | 0.3586 |

Abbreviations: R, right; L, left; p, proximal territory; m, middle territory; d, distal territory; MCA, middle cerebral arteries; ACA, anterior cerebral arteries; PCA, posterior cerebral arteries.

**TABLE S6.** Comparison of global functional connectivity density (gFCD) maps among healthy controls (HCs), Parkinson's disease with normal cognition (PD-NC), and Parkinson's disease with mild cognitive impairment (PD-MCI) groups based on the AAL3 atlas. The non-parametric Kruskal-Wallis test was used for comparisons among the three groups, with FDR-corrected significant results indicated in bold. Region numbering and anatomical labels were aligned with the original AAL3 atlas without modification. Chi-sq: Chi-square; df: degree of freedom.

| Label in AAL3 | Chi-sq | df | p value | FDR Corrected p value |
| --- | --- | --- | --- | --- |
| 1 Precentral_L 1 | 9.9777 | 2 | 0.0068 | 0.1467 |
| 2 Precentral_R 2 | 10.7949 | 2 | 0.0045 | 0.1389 |
| 3 Frontal_Sup_2_L 3 | 8.8667 | 2 | 0.0119 | 0.1561 |
| 4 Frontal_Sup_2_R 4 | 5.3958 | 2 | 0.0673 | 0.2197 |
| 5 Frontal_Mid_2_L 5 | 9.9496 | 2 | 0.0069 | 0.1467 |
| 6 Frontal_Mid_2_R 6 | 6.7833 | 2 | 0.0337 | 0.2009 |
| 7 Frontal_Inf_Oper_L 7 | 1.8087 | 2 | 0.4048 | 0.5354 |
| 8 Frontal_Inf_Oper_R 8 | 0.4344 | 2 | 0.8048 | 0.8414 |
| 9 Frontal_Inf_Tri_L 9 | 2.8378 | 2 | 0.242 | 0.4031 |
| 10 Frontal_Inf_Tri_R 10 | 1.3634 | 2 | 0.5057 | 0.6028 |
| 11 Frontal_Inf_Orb_2_L 11 | 0.5172 | 2 | 0.7721 | 0.8207 |
| 12 Frontal_Inf_Orb_2_R 12 | 0.036 | 2 | 0.9822 | 0.9875 |
| 13 Rolandic_Oper_L 13 | 1.5397 | 2 | 0.4631 | 0.568 |
| 14 Rolandic_Oper_R 14 | 2.2723 | 2 | 0.321 | 0.4688 |
| 15 Supp_Motor_Area_L 15 | 0.5239 | 2 | 0.7695 | 0.8207 |
| 16 Supp_Motor_Area_R 16 | 0.7568 | 2 | 0.685 | 0.7638 |
| 17 Olfactory_L 17 | 6.3388 | 2 | 0.042 | 0.2015 |
| 18 Olfactory_R 18 | 1.165 | 2 | 0.5585 | 0.6383 |
| 19 Frontal_Sup_Medial_L 19 | 7.7259 | 2 | 0.021 | 0.1904 |
| 20 Frontal_Sup_Medial_R 20 | 5.4905 | 2 | 0.0642 | 0.2189 |
| 21 Frontal_Med_Orb_L 21 | 6.2065 | 2 | 0.0449 | 0.2015 |
| 22 Frontal_Med_Orb_R 22 | 5.2055 | 2 | 0.0741 | 0.2301 |
| 23 Rectus_L 23 | 1.7817 | 2 | 0.4103 | 0.5354 |
| 24 Rectus_R 24 | 1.6763 | 2 | 0.4325 | 0.5451 |
| 25 OFCmed_L 25 | 0.8414 | 2 | 0.6566 | 0.7367 |
| 26 OFCmed_R 26 | 0.5966 | 2 | 0.7421 | 0.8032 |
| 27 OFCant_L 27 | 7.0834 | 2 | 0.029 | 0.1977 |
| 28 OFCant_R 28 | 3.162 | 2 | 0.2058 | 0.3817 |
| 29 OFCpost_L 29 | 0.6289 | 2 | 0.7302 | 0.795 |
| 30 OFCpost_R 30 | 1.2271 | 2 | 0.5414 | 0.6305 |
| 31 OFClat_L 31 | 1.2601 | 2 | 0.5326 | 0.6242 |
| 32 OFClat_R 32 | 5.6683 | 2 | 0.0588 | 0.2155 |
| 33 Insula_L 33 | 0.3315 | 2 | 0.8473 | 0.8661 |
| 34 Insula_R 34 | 0.4355 | 2 | 0.8043 | 0.8414 |
| 37 Cingulate_Mid_L 37 | 0.3408 | 2 | 0.8433 | 0.8661 |
| 38 Cingulate_Mid_R 38 | 2.25 | 2 | 0.3246 | 0.4704 |
| 39 Cingulate_Post_L 39 | 4.2548 | 2 | 0.1191 | 0.2923 |
| 40 Cingulate_Post_R 40 | 4.3378 | 2 | 0.1143 | 0.2881 |
| 41 Hippocampus_L 41 | 4.0131 | 2 | 0.1345 | 0.3129 |
| 42 Hippocampus_R 42 | 6.1434 | 2 | 0.0463 | 0.2015 |
| 43 ParaHippocampal_L 43 | 2.7126 | 2 | 0.2576 | 0.4122 |
| 44 ParaHippocampal_R 44 | 2.3196 | 2 | 0.3135 | 0.4615 |
| 45 Amygdala_L 45 | 2.3733 | 2 | 0.3052 | 0.4604 |
| 46 Amygdala_R 46 | 4.6727 | 2 | 0.0967 | 0.2505 |
| 47 Calcarine_L 47 | 0.5069 | 2 | 0.7761 | 0.8207 |
| 48 Calcarine_R 48 | 0.7386 | 2 | 0.6912 | 0.7662 |
| 49 Cuneus_L 49 | 2.0836 | 2 | 0.3528 | 0.4919 |
| 50 Cuneus_R 50 | 3.7084 | 2 | 0.1566 | 0.3237 |
| 51 Lingual_L 51 | 3.0604 | 2 | 0.2165 | 0.3944 |
| 52 Lingual_R 52 | 3.4487 | 2 | 0.1783 | 0.3453 |
| 53 Occipital_Sup_L 53 | 7.2577 | 2 | 0.0265 | 0.1977 |
| 54 Occipital_Sup_R 54 | 5.0503 | 2 | 0.08 | 0.2301 |
| 55 Occipital_Mid_L 55 | 2.5006 | 2 | 0.2864 | 0.4466 |
| 56 Occipital_Mid_R 56 | 2.068 | 2 | 0.3556 | 0.4919 |
| 57 Occipital_Inf_L 57 | 12.5811 | 2 | 1.90E-03 | 0.1137 |
| 58 Occipital_Inf_R 58 | 12.9244 | 2 | 0.0016 | 0.1137 |
| 59 Fusiform_L 59 | 4.8262 | 2 | 0.0895 | 0.2388 |
| 60 Fusiform_R 60 | 5.1596 | 2 | 0.0758 | 0.2301 |
| 61 Postcentral_L 61 | 9.721 | 2 | 0.0077 | 0.1467 |
| 62 Postcentral_R 62 | 8.0464 | 2 | 0.0179 | 0.1904 |
| 63 Parietal_Sup_L 63 | 2.8253 | 2 | 0.2435 | 0.4031 |
| 64 Parietal_Sup_R 64 | 1.681 | 2 | 0.4315 | 0.5451 |
| 65 Parietal_Inf_L 65 | 4.744 | 2 | 0.0933 | 0.2452 |
| 66 Parietal_Inf_R 66 | 1.7393 | 2 | 0.4191 | 0.539 |
| 67 SupraMarginal_L 67 | 1.6464 | 2 | 0.439 | 0.5495 |
| 68 SupraMarginal_R 68 | 2.7744 | 2 | 0.2498 | 0.4031 |
| 69 Angular_L 69 | 5.5911 | 2 | 0.0611 | 0.2155 |
| 70 Angular_R 70 | 8.3336 | 2 | 0.0155 | 0.1826 |
| 71 Precuneus_L 71 | 1.263 | 2 | 0.5318 | 0.6242 |
| 72 Precuneus_R 72 | 2.0151 | 2 | 0.3651 | 0.5014 |
| 73 Paracentral_Lobule_L 73 | 2.4323 | 2 | 0.2964 | 0.4544 |
| 74 Paracentral_Lobule_R 74 | 2.9118 | 2 | 0.2332 | 0.4031 |
| 75 Caudate_L 75 | 4.8831 | 2 | 0.087 | 0.2388 |
| 76 Caudate_R 76 | 0.2601 | 2 | 0.8781 | 0.8926 |
| 77 Putamen_L 77 | 1.3554 | 2 | 0.5078 | 0.6028 |
| 78 Putamen_R 78 | 5.0733 | 2 | 0.0791 | 0.2301 |
| 79 Pallidum_L 79 | 6.1215 | 2 | 0.0469 | 0.2015 |
| 80 Pallidum_R 80 | 1.8321 | 2 | 0.4001 | 0.5354 |
| 83 Heschl_L 83 | 2.8104 | 2 | 0.2453 | 0.4031 |
| 84 Heschl_R 84 | 5.5596 | 2 | 0.0621 | 0.2155 |
| 85 Temporal_Sup_L 85 | 9.0639 | 2 | 0.0108 | 0.1523 |
| 86 Temporal_Sup_R 86 | 7.1488 | 2 | 0.028 | 0.1977 |
| 87 Temporal_Pole_Sup_L 87 | 6.1111 | 2 | 0.0471 | 0.2015 |
| 88 Temporal_Pole_Sup_R 88 | 3.4496 | 2 | 0.1782 | 0.3453 |
| 89 Temporal_Mid_L 89 | 2.7813 | 2 | 0.2489 | 0.4031 |
| 90 Temporal_Mid_R 90 | 0.6936 | 2 | 0.707 | 0.7789 |
| 91 Temporal_Pole_Mid_L 91 | 2.0682 | 2 | 0.3555 | 0.4919 |
| 92 Temporal_Pole_Mid_R 92 | 3.8286 | 2 | 0.1474 | 0.3193 |
| 93 Temporal_Inf_L 93 | 0.0679 | 2 | 0.9666 | 0.9772 |
| 94 Temporal_Inf_R 94 | 2.6624 | 2 | 0.2642 | 0.419 |
| 95 Cerebellum_Crus1_L 95 | 5.7146 | 2 | 0.0574 | 0.2155 |
| 96 Cerebellum_Crus1_R 96 | 1.9296 | 2 | 0.3811 | 0.5194 |
| 97 Cerebellum_Crus2_L 97 | 3.7937 | 2 | 0.15 | 0.3193 |
| 98 Cerebellum_Crus2_R 98 | 1.8007 | 2 | 0.4064 | 0.5354 |
| 99 Cerebellum_3_L 99 | 1.191 | 2 | 0.5513 | 0.6356 |
| 100 Cerebellum_3_R 100 | 2.8293 | 2 | 0.243 | 0.4031 |
| 101 Cerebellum_4_5_L 101 | 5.9062 | 2 | 0.0522 | 0.2087 |
| 102 Cerebellum_4_5_R 102 | 3.9855 | 2 | 0.1363 | 0.3129 |
| 103 Cerebellum_6_L 103 | 5.1326 | 2 | 0.0768 | 0.2301 |
| 104 Cerebellum_6_R 104 | 7.646 | 2 | 0.0219 | 0.1904 |
| 105 Cerebellum_7b_L 105 | 3.7586 | 2 | 0.1527 | 0.3193 |
| 106 Cerebellum_7b_R 106 | 2.881 | 2 | 0.2368 | 0.4031 |
| 107 Cerebellum_8_L 107 | 7.8268 | 2 | 0.02 | 0.1904 |
| 108 Cerebellum_8_R 108 | 7.093 | 2 | 0.0288 | 0.1977 |
| 109 Cerebellum_9_L 109 | 6.9369 | 2 | 0.0312 | 0.1977 |
| 110 Cerebellum_9_R 110 | 9.4448 | 2 | 0.0089 | 0.1487 |
| 111 Cerebellum_10_L 111 | 4.0309 | 2 | 0.1333 | 0.3129 |
| 112 Cerebellum_10_R 112 | 6.6981 | 2 | 0.0351 | 0.2015 |
| 113 Vermis_1_2 113 | 2.3301 | 2 | 0.3119 | 0.4615 |
| 114 Vermis_3 114 | 1.7856 | 2 | 0.4095 | 0.5354 |
| 115 Vermis_4_5 115 | 7.5651 | 2 | 0.0228 | 0.1904 |
| 116 Vermis_6 116 | 4.8471 | 2 | 0.0886 | 0.2388 |
| 117 Vermis_7 117 | 5.0744 | 2 | 0.0791 | 0.2301 |
| 118 Vermis_8 118 | 4.0633 | 2 | 0.1311 | 0.3129 |
| 119 Vermis_9 119 | 5.9245 | 2 | 0.0517 | 0.2087 |
| 120 Vermis_10 120 | 2.1062 | 2 | 0.3489 | 0.4919 |
| 121 Thal_AV_L 121 | 6.2099 | 2 | 0.0448 | 0.2015 |
| 122 Thal_AV_R 122 | 4.8374 | 2 | 0.089 | 0.2388 |
| 123 Thal_LP_L 123 | 2.4005 | 2 | 0.3011 | 0.4579 |
| 124 Thal_LP_R 124 | 3.2708 | 2 | 0.1949 | 0.3659 |
| 125 Thal_VA_L 125 | 4.5664 | 2 | 0.102 | 0.2606 |
| 126 Thal_VA_R 126 | 2.6361 | 2 | 0.2677 | 0.4209 |
| 127 Thal_VL_L 127 | 3.7664 | 2 | 0.1521 | 0.3193 |
| 128 Thal_VL_R 128 | 5.3749 | 2 | 0.0681 | 0.2197 |
| 129 Thal_VPL_L 129 | 2.3486 | 2 | 0.309 | 0.4615 |
| 130 Thal_VPL_R 130 | 4.2703 | 2 | 0.1182 | 0.2923 |
| 131 Thal_IL_L 131 | 5.804 | 2 | 0.0549 | 0.2105 |
| 132 Thal_IL_R 132 | 3.1456 | 2 | 0.2075 | 0.3817 |
| 133 Thal_Re_L 133 | 0.637 | 2 | 0.7272 | 0.795 |
| 134 Thal_Re_R 134 | 6.4787 | 2 | 0.0392 | 0.2015 |
| 135 Thal_MDm_L 135 | 7.0007 | 2 | 0.0302 | 0.1977 |
| 136 Thal_MDm_R 136 | 3.9647 | 2 | 0.1377 | 0.3129 |
| 137 Thal_MDl_L 137 | 5.4299 | 2 | 0.0662 | 0.2197 |
| 138 Thal_MDl_R 138 | 5.5585 | 2 | 0.0621 | 0.2155 |
| 139 Thal_LGN_L 139 | 5.8396 | 2 | 0.0539 | 0.2105 |
| 140 Thal_LGN_R 140 | 2.8605 | 2 | 0.2392 | 0.4031 |
| 141 Thal_MGN_L 141 | 3.3902 | 2 | 0.1836 | 0.3519 |
| 142 Thal_MGN_R 142 | 3.5094 | 2 | 0.173 | 0.3422 |
| 143 Thal_PuI_L 143 | 7.8472 | 2 | 0.0198 | 0.1904 |
| 144 Thal_PuI_R 144 | 2.8971 | 2 | 0.2349 | 0.4031 |
| 145 Thal_PuM_L 145 | 13.7058 | 2 | 0.0011 | 0.1137 |
| 146 Thal_PuM_R 146 | 6.2894 | 2 | 0.0431 | 0.2015 |
| 147 Thal_PuA_L 147 | 5.0629 | 2 | 0.0795 | 0.2301 |
| 148 Thal_PuA_R 148 | 0.3957 | 2 | 0.8205 | 0.8495 |
| 149 Thal_PuL_L 149 | 0.0216 | 2 | 0.9892 | 0.9892 |
| 150 Thal_PuL_R 150 | 1.7262 | 2 | 0.4219 | 0.539 |
| 151 ACC_sub_L 151 | 1.556 | 2 | 0.4593 | 0.5672 |
| 152 ACC_sub_R 152 | 1.4241 | 2 | 0.4906 | 0.5939 |
| 153 ACC_pre_L 153 | 8.2861 | 2 | 0.0159 | 0.1826 |
| 154 ACC_pre_R 154 | 6.6094 | 2 | 0.0367 | 0.2015 |
| 155 ACC_sup_L 155 | 3.8542 | 2 | 0.1456 | 0.3193 |
| 156 ACC_sup_R 156 | 3.6226 | 2 | 0.1634 | 0.3269 |
| 157 N_Acc_L 157 | 2.8994 | 2 | 0.2346 | 0.4031 |
| 158 N_Acc_R 158 | 0.3926 | 2 | 0.8218 | 0.8495 |
| 159 VTA_L 159 | 1.4809 | 2 | 0.4769 | 0.5811 |
| 160 VTA_R 160 | 1.1085 | 2 | 0.5745 | 0.6525 |
| 161 SN_pc_L 161 | 1.3788 | 2 | 0.5019 | 0.6028 |
| 162 SN_pc_R 162 | 4.1419 | 2 | 0.1261 | 0.3052 |
| 163 SN_pr_L 163 | 1.8385 | 2 | 0.3988 | 0.5354 |
| 164 SN_pr_R 164 | 4.8975 | 2 | 0.0864 | 0.2388 |
| 165 Red_N_L 165 | 6.1218 | 2 | 0.0468 | 0.2015 |
| 166 Red_N_R 166 | 5.9845 | 2 | 0.0502 | 0.2087 |
| 167 LC_L 167 | 0.5513 | 2 | 0.7591 | 0.8168 |
| 168 LC_R 168 | 1.186 | 2 | 0.5527 | 0.6356 |
| 169 Raphe_D 169 | 9.2719 | 2 | 0.0097 | 0.1487 |
| 170 Raphe_M 170 | 2.4491 | 2 | 0.2939 | 0.4544 |

**TABLE S7.** Comparison of global functional connectivity density (gFCD) maps among healthy controls (HCs), Parkinson's disease with normal cognition (PD-NC), and Parkinson's disease with mild cognitive impairment (PD-MCI) groups based on the arterial territory template. The non-parametric Kruskal-Wallis test was used for comparisons among the three groups, with FDR-corrected significant results indicated in bold. Chi-sq: Chi-square; df: degree of freedom.

| Arterial Territories | Chi-sq | df | p value | FDR Corrected p value |
| --- | --- | --- | --- | --- |
| RACA_p | 6.9575 | 2 | 0.0308 | 0.1977 |
| RACA_m | 3.7746 | 2 | 0.1515 | 0.3193 |
| RACA_d | 3.3664 | 2 | 0.1858 | 0.3524 |
| RMCA_p | 1.6226 | 2 | 0.4443 | 0.5524 |
| RMCA_m | 3.8477 | 2 | 0.146 | 0.3193 |
| RMCA_d | 2.7757 | 2 | 0.2496 | 0.4031 |
| RPCA_p | 11.7997 | 2 | 0.0027 | 0.126 |
| RPCA_m | 2.2186 | 2 | 0.3298 | 0.4741 |
| RPCA_d | 2.9278 | 2 | 0.2313 | 0.4031 |
| LACA_p | 11.3488 | 2 | 0.0034 | 0.1263 |
| LACA_m | 6.7714 | 2 | 0.0339 | 0.2009 |
| LACA_d | 1.0357 | 2 | 0.5958 | 0.6726 |
| LMCA_p | 1.7386 | 2 | 0.4193 | 0.539 |
| LMCA_m | 6.343 | 2 | 0.0419 | 0.2015 |
| LMCA_d | 3.661 | 2 | 0.1603 | 0.3269 |
| LPCA_p | 9.6628 | 2 | 0.008 | 0.1467 |
| LPCA_m | 3.6246 | 2 | 0.1633 | 0.3269 |
| LPCA_d | 2.1455 | 2 | 0.3421 | 0.4879 |

Abbreviations: R, right; L, left; p, proximal territory; m, middle territory; d, distal territory; MCA, middle cerebral arteries; ACA, anterior cerebral arteries; PCA, posterior cerebral arteries.

**TABLE S8.** Comparison of local functional connectivity density (lFCD) maps among healthy controls (HCs), Parkinson's disease with normal cognition (PD-NC), and Parkinson's disease with mild cognitive impairment (PD-MCI) groups based on the AAL3 atlas. The non-parametric Kruskal-Wallis test was used for comparisons among the three groups, with FDR-corrected significant results indicated in bold. Region numbering and anatomical labels were aligned with the original AAL3 atlas without modification. Chi-sq: Chi-square; df: degree of freedom.

| Label in AAL3 | Chi-sq | df | p value | FDR Corrected p value |
| --- | --- | --- | --- | --- |
| 1 Precentral_L 1 | 9.8097 | 2 | 0.0074 | 0.1505 |
| 2 Precentral_R 2 | 7.9379 | 2 | 0.0189 | 0.199 |
| 3 Frontal_Sup_2_L 3 | 11.4382 | 2 | 0.0033 | 0.1505 |
| 4 Frontal_Sup_2_R 4 | 5.037 | 2 | 0.0806 | 0.2513 |
| 5 Frontal_Mid_2_L 5 | 13.0914 | 2 | 0.0014 | 0.1505 |
| 6 Frontal_Mid_2_R 6 | 3.4731 | 2 | 0.1761 | 0.3877 |
| 7 Frontal_Inf_Oper_L 7 | 3.5705 | 2 | 0.1678 | 0.3811 |
| 8 Frontal_Inf_Oper_R 8 | 0.5045 | 2 | 0.7771 | 0.8265 |
| 9 Frontal_Inf_Tri_L 9 | 6.5663 | 2 | 0.0375 | 0.199 |
| 10 Frontal_Inf_Tri_R 10 | 0.2875 | 2 | 0.8661 | 0.8879 |
| 11 Frontal_Inf_Orb_2_L 11 | 2.4368 | 2 | 0.2957 | 0.4963 |
| 12 Frontal_Inf_Orb_2_R 12 | 0.1465 | 2 | 0.9294 | 0.9294 |
| 13 Rolandic_Oper_L 13 | 1.5776 | 2 | 0.4544 | 0.5847 |
| 14 Rolandic_Oper_R 14 | 2.4948 | 2 | 0.2873 | 0.4963 |
| 15 Supp_Motor_Area_L 15 | 0.6995 | 2 | 0.7048 | 0.7754 |
| 16 Supp_Motor_Area_R 16 | 0.7435 | 2 | 0.6895 | 0.7736 |
| 17 Olfactory_L 17 | 4.9677 | 2 | 0.0834 | 0.2558 |
| 18 Olfactory_R 18 | 0.3246 | 2 | 0.8502 | 0.8788 |
| 19 Frontal_Sup_Medial_L 19 | 8.7998 | 2 | 0.0123 | 0.1883 |
| 20 Frontal_Sup_Medial_R 20 | 7.1168 | 2 | 0.0285 | 0.199 |
| 21 Frontal_Med_Orb_L 21 | 7.5869 | 2 | 0.0225 | 0.199 |
| 22 Frontal_Med_Orb_R 22 | 6.3548 | 2 | 0.0417 | 0.199 |
| 23 Rectus_L 23 | 2.2198 | 2 | 0.3296 | 0.4963 |
| 24 Rectus_R 24 | 2.3853 | 2 | 0.3034 | 0.4963 |
| 25 OFCmed_L 25 | 1.8666 | 2 | 0.3933 | 0.5441 |
| 26 OFCmed_R 26 | 2.3599 | 2 | 0.3073 | 0.4963 |
| 27 OFCant_L 27 | 3.8492 | 2 | 0.1459 | 0.3678 |
| 28 OFCant_R 28 | 1.8335 | 2 | 0.3998 | 0.5462 |
| 29 OFCpost_L 29 | 0.1522 | 2 | 0.9267 | 0.9294 |
| 30 OFCpost_R 30 | 0.189 | 2 | 0.9098 | 0.9198 |
| 31 OFClat_L 31 | 1.0181 | 2 | 0.6011 | 0.7038 |
| 32 OFClat_R 32 | 3.7489 | 2 | 0.1534 | 0.3702 |
| 33 Insula_L 33 | 0.2818 | 2 | 0.8686 | 0.8879 |
| 34 Insula_R 34 | 0.6353 | 2 | 0.7279 | 0.7786 |
| 37 Cingulate_Mid_L 37 | 1.6562 | 2 | 0.4369 | 0.5701 |
| 38 Cingulate_Mid_R 38 | 1.0766 | 2 | 0.5837 | 0.6968 |
| 39 Cingulate_Post_L 39 | 5.497 | 2 | 0.064 | 0.2349 |
| 40 Cingulate_Post_R 40 | 6.0381 | 2 | 0.0488 | 0.1997 |
| 41 Hippocampus_L 41 | 3.8223 | 2 | 0.1479 | 0.3678 |
| 42 Hippocampus_R 42 | 7.7651 | 2 | 0.0206 | 0.199 |
| 43 ParaHippocampal_L 43 | 3.8665 | 2 | 0.1447 | 0.3678 |
| 44 ParaHippocampal_R 44 | 5.5135 | 2 | 0.0635 | 0.2349 |
| 45 Amygdala_L 45 | 0.3539 | 2 | 0.8378 | 0.8779 |
| 46 Amygdala_R 46 | 1.7547 | 2 | 0.4159 | 0.5505 |
| 47 Calcarine_L 47 | 2.2799 | 2 | 0.3198 | 0.4963 |
| 48 Calcarine_R 48 | 3.8454 | 2 | 0.1462 | 0.3678 |
| 49 Cuneus_L 49 | 5.2408 | 2 | 0.0728 | 0.2349 |
| 50 Cuneus_R 50 | 6.6246 | 2 | 0.0364 | 0.199 |
| 51 Lingual_L 51 | 1.9721 | 2 | 0.373 | 0.5304 |
| 52 Lingual_R 52 | 2.2762 | 2 | 0.3204 | 0.4963 |
| 53 Occipital_Sup_L 53 | 9.6233 | 2 | 0.0081 | 0.1505 |
| 54 Occipital_Sup_R 54 | 7.2059 | 2 | 0.0272 | 0.199 |
| 55 Occipital_Mid_L 55 | 2.8986 | 2 | 0.2347 | 0.4499 |
| 56 Occipital_Mid_R 56 | 1.7593 | 2 | 0.4149 | 0.5505 |
| 57 Occipital_Inf_L 57 | 6.7262 | 2 | 3.46E-02 | 0.199 |
| 58 Occipital_Inf_R 58 | 7.5933 | 2 | 0.0224 | 0.199 |
| 59 Fusiform_L 59 | 2.3653 | 2 | 0.3065 | 0.4963 |
| 60 Fusiform_R 60 | 1.4627 | 2 | 0.4813 | 0.6065 |
| 61 Postcentral_L 61 | 10.0492 | 2 | 0.0066 | 0.1505 |
| 62 Postcentral_R 62 | 6.8312 | 2 | 0.0329 | 0.199 |
| 63 Parietal_Sup_L 63 | 2.2543 | 2 | 0.324 | 0.4963 |
| 64 Parietal_Sup_R 64 | 1.7944 | 2 | 0.4077 | 0.5503 |
| 65 Parietal_Inf_L 65 | 0.4548 | 2 | 0.7966 | 0.8424 |
| 66 Parietal_Inf_R 66 | 0.7925 | 2 | 0.6728 | 0.7642 |
| 67 SupraMarginal_L 67 | 2.7712 | 2 | 0.2502 | 0.465 |
| 68 SupraMarginal_R 68 | 0.9153 | 2 | 0.6328 | 0.7315 |
| 69 Angular_L 69 | 6.1622 | 2 | 0.0459 | 0.199 |
| 70 Angular_R 70 | 8.4642 | 2 | 0.0145 | 0.199 |
| 71 Precuneus_L 71 | 0.7022 | 2 | 0.7039 | 0.7754 |
| 72 Precuneus_R 72 | 2.3255 | 2 | 0.3126 | 0.4963 |
| 73 Paracentral_Lobule_L 73 | 2.7776 | 2 | 0.2494 | 0.465 |
| 74 Paracentral_Lobule_R 74 | 3.8645 | 2 | 0.1448 | 0.3678 |
| 75 Caudate_L 75 | 10.1666 | 2 | 0.0062 | 0.1505 |
| 76 Caudate_R 76 | 2.2835 | 2 | 0.3193 | 0.4963 |
| 77 Putamen_L 77 | 0.8099 | 2 | 0.667 | 0.7623 |
| 78 Putamen_R 78 | 1.0072 | 2 | 0.6044 | 0.7038 |
| 79 Pallidum_L 79 | 1.9631 | 2 | 0.3747 | 0.5304 |
| 80 Pallidum_R 80 | 0.6707 | 2 | 0.7151 | 0.7754 |
| 83 Heschl_L 83 | 3.2966 | 2 | 0.1924 | 0.4022 |
| 84 Heschl_R 84 | 5.275 | 2 | 0.0715 | 0.2349 |
| 85 Temporal_Sup_L 85 | 9.9106 | 2 | 0.007 | 0.1505 |
| 86 Temporal_Sup_R 86 | 8.1022 | 2 | 0.0174 | 0.199 |
| 87 Temporal_Pole_Sup_L 87 | 6.1197 | 2 | 0.0469 | 0.199 |
| 88 Temporal_Pole_Sup_R 88 | 5.839 | 2 | 0.054 | 0.2158 |
| 89 Temporal_Mid_L 89 | 6.5913 | 2 | 0.037 | 0.199 |
| 90 Temporal_Mid_R 90 | 1.1448 | 2 | 0.5642 | 0.6829 |
| 91 Temporal_Pole_Mid_L 91 | 3.2633 | 2 | 0.1956 | 0.4044 |
| 92 Temporal_Pole_Mid_R 92 | 3.6679 | 2 | 0.1598 | 0.3769 |
| 93 Temporal_Inf_L 93 | 1.0827 | 2 | 0.582 | 0.6968 |
| 94 Temporal_Inf_R 94 | 5.6435 | 2 | 0.0595 | 0.2329 |
| 95 Cerebellum_Crus1_L 95 | 6.5205 | 2 | 0.0384 | 0.199 |
| 96 Cerebellum_Crus1_R 96 | 2.9503 | 2 | 0.2287 | 0.4455 |
| 97 Cerebellum_Crus2_L 97 | 4.8411 | 2 | 0.0889 | 0.2636 |
| 98 Cerebellum_Crus2_R 98 | 2.2503 | 2 | 0.3246 | 0.4963 |
| 99 Cerebellum_3_L 99 | 1.028 | 2 | 0.5981 | 0.7038 |
| 100 Cerebellum_3_R 100 | 3.1655 | 2 | 0.2054 | 0.4163 |
| 101 Cerebellum_4_5_L 101 | 4.8101 | 2 | 0.0903 | 0.2636 |
| 102 Cerebellum_4_5_R 102 | 1.5913 | 2 | 0.4513 | 0.5847 |
| 103 Cerebellum_6_L 103 | 4.134 | 2 | 0.1266 | 0.3469 |
| 104 Cerebellum_6_R 104 | 5.2682 | 2 | 0.0718 | 0.2349 |
| 105 Cerebellum_7b_L 105 | 6.2018 | 2 | 0.045 | 0.199 |
| 106 Cerebellum_7b_R 106 | 5.4218 | 2 | 0.0665 | 0.2349 |
| 107 Cerebellum_8_L 107 | 6.0899 | 2 | 0.0476 | 0.199 |
| 108 Cerebellum_8_R 108 | 5.3999 | 2 | 0.0672 | 0.2349 |
| 109 Cerebellum_9_L 109 | 3.7846 | 2 | 0.1507 | 0.3698 |
| 110 Cerebellum_9_R 110 | 5.2853 | 2 | 0.0712 | 0.2349 |
| 111 Cerebellum_10_L 111 | 3.542 | 2 | 0.1702 | 0.3818 |
| 112 Cerebellum_10_R 112 | 5.288 | 2 | 0.0711 | 0.2349 |
| 113 Vermis_1_2 113 | 2.0518 | 2 | 0.3585 | 0.5277 |
| 114 Vermis_3 114 | 2.2159 | 2 | 0.3302 | 0.4963 |
| 115 Vermis_4_5 115 | 6.9427 | 2 | 0.0311 | 0.199 |
| 116 Vermis_6 116 | 3.4396 | 2 | 0.1791 | 0.3877 |
| 117 Vermis_7 117 | 4.5408 | 2 | 0.1033 | 0.2923 |
| 118 Vermis_8 118 | 2.5901 | 2 | 0.2739 | 0.4941 |
| 119 Vermis_9 119 | 2.9392 | 2 | 0.23 | 0.4455 |
| 120 Vermis_10 120 | 1.0655 | 2 | 0.587 | 0.6968 |
| 121 Thal_AV_L 121 | 6.4741 | 2 | 0.0393 | 0.199 |
| 122 Thal_AV_R 122 | 2.3343 | 2 | 0.3113 | 0.4963 |
| 123 Thal_LP_L 123 | 6.2316 | 2 | 0.0443 | 0.199 |
| 124 Thal_LP_R 124 | 9.6127 | 2 | 0.0082 | 0.1505 |
| 125 Thal_VA_L 125 | 4.8284 | 2 | 0.0894 | 0.2636 |
| 126 Thal_VA_R 126 | 1.2639 | 2 | 0.5315 | 0.6564 |
| 127 Thal_VL_L 127 | 3.1607 | 2 | 0.2059 | 0.4163 |
| 128 Thal_VL_R 128 | 2.5227 | 2 | 0.2833 | 0.4963 |
| 129 Thal_VPL_L 129 | 2.5968 | 2 | 0.273 | 0.4941 |
| 130 Thal_VPL_R 130 | 0.6382 | 2 | 0.7268 | 0.7786 |
| 131 Thal_IL_L 131 | 2.0156 | 2 | 0.365 | 0.5288 |
| 132 Thal_IL_R 132 | 0.3283 | 2 | 0.8486 | 0.8788 |
| 133 Thal_Re_L 133 | 6.5873 | 2 | 0.0371 | 0.199 |
| 134 Thal_Re_R 134 | 0.2699 | 2 | 0.8738 | 0.8882 |
| 135 Thal_MDm_L 135 | 1.976 | 2 | 0.3723 | 0.5304 |
| 136 Thal_MDm_R 136 | 1.3405 | 2 | 0.5116 | 0.636 |
| 137 Thal_MDl_L 137 | 1.4688 | 2 | 0.4798 | 0.6065 |
| 138 Thal_MDl_R 138 | 2.2773 | 2 | 0.3203 | 0.4963 |
| 139 Thal_LGN_L 139 | 6.271 | 2 | 0.0435 | 0.199 |
| 140 Thal_LGN_R 140 | 3.3675 | 2 | 0.1857 | 0.3973 |
| 141 Thal_MGN_L 141 | 7.4609 | 2 | 0.024 | 0.199 |
| 142 Thal_MGN_R 142 | 1.8287 | 2 | 0.4008 | 0.5462 |
| 143 Thal_PuI_L 143 | 8.9706 | 2 | 0.0113 | 0.1883 |
| 144 Thal_PuI_R 144 | 2.6325 | 2 | 0.2681 | 0.4934 |
| 145 Thal_PuM_L 145 | 6.7746 | 2 | 0.0338 | 0.199 |
| 146 Thal_PuM_R 146 | 3.0392 | 2 | 0.2188 | 0.4355 |
| 147 Thal_PuA_L 147 | 3.5824 | 2 | 0.1668 | 0.3811 |
| 148 Thal_PuA_R 148 | 0.9049 | 2 | 0.6361 | 0.7315 |
| 149 Thal_PuL_L 149 | 2.3155 | 2 | 0.3142 | 0.4963 |
| 150 Thal_PuL_R 150 | 2.5496 | 2 | 0.2795 | 0.4963 |
| 151 ACC_sub_L 151 | 1.9413 | 2 | 0.3788 | 0.5321 |
| 152 ACC_sub_R 152 | 1.5279 | 2 | 0.4658 | 0.5952 |
| 153 ACC_pre_L 153 | 7.2343 | 2 | 0.0269 | 0.199 |
| 154 ACC_pre_R 154 | 6.6968 | 2 | 0.0351 | 0.199 |
| 155 ACC_sup_L 155 | 3.2999 | 2 | 0.1921 | 0.4022 |
| 156 ACC_sup_R 156 | 3.0271 | 2 | 0.2201 | 0.4355 |
| 157 N_Acc_L 157 | 5.1516 | 2 | 0.0761 | 0.2414 |
| 158 N_Acc_R 158 | 1.9242 | 2 | 0.3821 | 0.5326 |
| 159 VTA_L 159 | 0.7736 | 2 | 0.6792 | 0.7667 |
| 160 VTA_R 160 | 1.3804 | 2 | 0.5015 | 0.6277 |
| 161 SN_pc_L 161 | 1.7192 | 2 | 0.4233 | 0.5564 |
| 162 SN_pc_R 162 | 3.4539 | 2 | 0.1778 | 0.3877 |
| 163 SN_pr_L 163 | 3.6395 | 2 | 0.1621 | 0.3775 |
| 164 SN_pr_R 164 | 5.4247 | 2 | 0.0664 | 0.2349 |
| 165 Red_N_L 165 | 2.7954 | 2 | 0.2472 | 0.465 |
| 166 Red_N_R 166 | 2.2065 | 2 | 0.3318 | 0.4963 |
| 167 LC_L 167 | 0.6671 | 2 | 0.7164 | 0.7754 |
| 168 LC_R 168 | 0.3492 | 2 | 0.8398 | 0.8779 |
| 169 Raphe_D 169 | 4.3575 | 2 | 0.1132 | 0.3155 |
| 170 Raphe_M 170 | 0.6719 | 2 | 0.7147 | 0.7754 |

**TABLE S9.** Comparison of global functional connectivity density (lFCD) maps among healthy controls (HCs), Parkinson's disease with normal cognition (PD-NC), and Parkinson's disease with mild cognitive impairment (PD-MCI) groups based on the arterial territory template. The non-parametric Kruskal-Wallis test was used for comparisons among the three groups, with FDR-corrected significant results indicated in bold. Chi-sq: Chi-square; df: degree of freedom.

| Arterial Territories | Chi-sq | df | p value | FDR Corrected p value |
| --- | --- | --- | --- | --- |
| RACA_p | 7.9345 | 2 | 0.0189 | 0.199 |
| RACA_m | 6.2759 | 2 | 0.0434 | 0.199 |
| RACA_d | 4.1085 | 2 | 0.1282 | 0.3469 |
| RMCA_p | 2.0236 | 2 | 0.3636 | 0.5288 |
| RMCA_m | 2.5315 | 2 | 0.282 | 0.4963 |
| RMCA_d | 2.3132 | 2 | 0.3145 | 0.4963 |
| RPCA_p | 10.3749 | 2 | 0.0056 | 0.1505 |
| RPCA_m | 0.6899 | 2 | 0.7082 | 0.7754 |
| RPCA_d | 2.0896 | 2 | 0.3518 | 0.522 |
| LACA_p | 11.234 | 2 | 0.0036 | 0.1505 |
| LACA_m | 7.5335 | 2 | 0.0231 | 0.199 |
| LACA_d | 1.7844 | 2 | 0.4097 | 0.5503 |
| LMCA_p | 1.2326 | 2 | 0.5399 | 0.6579 |
| LMCA_m | 4.0496 | 2 | 0.132 | 0.352 |
| LMCA_d | 4.6326 | 2 | 0.0986 | 0.2836 |
| LPCA_p | 8.334 | 2 | 0.0155 | 0.199 |
| LPCA_m | 1.2457 | 2 | 0.5364 | 0.6579 |
| LPCA_d | 3.7295 | 2 | 0.1549 | 0.3702 |

Abbreviations: R, right; L, left; p, proximal territory; m, middle territory; d, distal territory; MCA, middle cerebral arteries; ACA, anterior cerebral arteries; PCA, posterior cerebral arteries.

**TABLE S10.** Comparison of long-range functional connectivity density (lrFCD) maps among healthy controls (HCs), Parkinson's disease with normal cognition (PD-NC), and Parkinson's disease with mild cognitive impairment (PD-MCI) groups based on the AAL3 atlas. The non-parametric Kruskal-Wallis test was used for comparisons among the three groups, with FDR-corrected significant results indicated in bold. Region numbering and anatomical labels were aligned with the original AAL3 atlas without modification. Chi-sq: Chi-square; df: degree of freedom.

| Label in AAL3 | Chi-sq | df | p value | FDR Corrected p value |
| --- | --- | --- | --- | --- |
| 1 Precentral_L 1 | 5.9724 | 2 | 0.0505 | 0.1858 |
| 2 Precentral_R 2 | 7.1361 | 2 | 0.0282 | 0.1575 |
| 3 Frontal_Sup_2_L 3 | 3.6109 | 2 | 0.1644 | 0.3437 |
| 4 Frontal_Sup_2_R 4 | 4.8655 | 2 | 0.0878 | 0.2412 |
| 5 Frontal_Mid_2_L 5 | 6.7055 | 2 | 0.035 | 0.1752 |
| 6 Frontal_Mid_2_R 6 | 5.6967 | 2 | 0.0579 | 0.1904 |
| 7 Frontal_Inf_Oper_L 7 | 1.7715 | 2 | 0.4124 | 0.527 |
| 8 Frontal_Inf_Oper_R 8 | 2.0072 | 2 | 0.3666 | 0.4983 |
| 9 Frontal_Inf_Tri_L 9 | 1.9937 | 2 | 0.369 | 0.4983 |
| 10 Frontal_Inf_Tri_R 10 | 2.1702 | 2 | 0.3379 | 0.4983 |
| 11 Frontal_Inf_Orb_2_L 11 | 0.0019 | 2 | 0.999 | 0.999 |
| 12 Frontal_Inf_Orb_2_R 12 | 0.0304 | 2 | 0.9849 | 0.9957 |
| 13 Rolandic_Oper_L 13 | 0.412 | 2 | 0.8138 | 0.87 |
| 14 Rolandic_Oper_R 14 | 1.236 | 2 | 0.539 | 0.6568 |
| 15 Supp_Motor_Area_L 15 | 0.6675 | 2 | 0.7162 | 0.7939 |
| 16 Supp_Motor_Area_R 16 | 0.0976 | 2 | 0.9524 | 0.9793 |
| 17 Olfactory_L 17 | 2.4145 | 2 | 0.299 | 0.4663 |
| 18 Olfactory_R 18 | 3.9399 | 2 | 0.1395 | 0.3092 |
| 19 Frontal_Sup_Medial_L 19 | 4.9091 | 2 | 0.0859 | 0.2412 |
| 20 Frontal_Sup_Medial_R 20 | 3.5748 | 2 | 0.1674 | 0.3461 |
| 21 Frontal_Med_Orb_L 21 | 0.8153 | 2 | 0.6652 | 0.7509 |
| 22 Frontal_Med_Orb_R 22 | 0.5488 | 2 | 0.76 | 0.8324 |
| 23 Rectus_L 23 | 0.097 | 2 | 0.9527 | 0.9793 |
| 24 Rectus_R 24 | 0.0118 | 2 | 0.9941 | 0.999 |
| 25 OFCmed_L 25 | 0.8612 | 2 | 0.6501 | 0.7384 |
| 26 OFCmed_R 26 | 1.0513 | 2 | 0.5912 | 0.7018 |
| 27 OFCant_L 27 | 1.9896 | 2 | 0.3698 | 0.4983 |
| 28 OFCant_R 28 | 0.2393 | 2 | 0.8873 | 0.9329 |
| 29 OFCpost_L 29 | 0.9918 | 2 | 0.609 | 0.7138 |
| 30 OFCpost_R 30 | 2.814 | 2 | 0.2449 | 0.4172 |
| 31 OFClat_L 31 | 1.4612 | 2 | 0.4816 | 0.6029 |
| 32 OFClat_R 32 | 4.5522 | 2 | 0.1027 | 0.2661 |
| 33 Insula_L 33 | 0.7002 | 2 | 0.7046 | 0.7858 |
| 34 Insula_R 34 | 0.2496 | 2 | 0.8827 | 0.9329 |
| 37 Cingulate_Mid_L 37 | 0.0838 | 2 | 0.959 | 0.9803 |
| 38 Cingulate_Mid_R 38 | 4.4777 | 2 | 0.1066 | 0.268 |
| 39 Cingulate_Post_L 39 | 2.0487 | 2 | 0.359 | 0.4983 |
| 40 Cingulate_Post_R 40 | 2.1347 | 2 | 0.3439 | 0.4983 |
| 41 Hippocampus_L 41 | 5.3659 | 2 | 0.0684 | 0.2007 |
| 42 Hippocampus_R 42 | 6.3225 | 2 | 0.0424 | 0.1752 |
| 43 ParaHippocampal_L 43 | 2.1071 | 2 | 0.3487 | 0.4983 |
| 44 ParaHippocampal_R 44 | 2.8904 | 2 | 0.2357 | 0.4117 |
| 45 Amygdala_L 45 | 4.0995 | 2 | 0.1288 | 0.2962 |
| 46 Amygdala_R 46 | 8.3303 | 2 | 0.0155 | 0.1495 |
| 47 Calcarine_L 47 | 4.0569 | 2 | 0.1315 | 0.2988 |
| 48 Calcarine_R 48 | 3.0461 | 2 | 0.218 | 0.4012 |
| 49 Cuneus_L 49 | 0.1268 | 2 | 0.9386 | 0.9757 |
| 50 Cuneus_R 50 | 2.6701 | 2 | 0.2631 | 0.4402 |
| 51 Lingual_L 51 | 8.4373 | 2 | 0.0147 | 0.1495 |
| 52 Lingual_R 52 | 6.3553 | 2 | 0.0417 | 0.1752 |
| 53 Occipital_Sup_L 53 | 2.6482 | 2 | 0.266 | 0.441 |
| 54 Occipital_Sup_R 54 | 2.4821 | 2 | 0.2891 | 0.4546 |
| 55 Occipital_Mid_L 55 | 2.0811 | 2 | 0.3533 | 0.4983 |
| 56 Occipital_Mid_R 56 | 3.5325 | 2 | 0.171 | 0.3493 |
| 57 Occipital_Inf_L 57 | **17.2775** | **2** | **2.00E-04** | **0.0326** |
| 58 Occipital_Inf_R 58 | 13.3232 | 2 | 0.0013 | 0.0516 |
| 59 Fusiform_L 59 | 5.2099 | 2 | 0.0739 | 0.2125 |
| 60 Fusiform_R 60 | 9.0639 | 2 | 0.0108 | 0.1495 |
| 61 Postcentral_L 61 | 6.2277 | 2 | 0.0444 | 0.1752 |
| 62 Postcentral_R 62 | 6.9627 | 2 | 0.0308 | 0.1665 |
| 63 Parietal_Sup_L 63 | 5.8981 | 2 | 0.0524 | 0.1872 |
| 64 Parietal_Sup_R 64 | 2.2238 | 2 | 0.3289 | 0.4961 |
| 65 Parietal_Inf_L 65 | 9.9857 | 2 | 0.0068 | 0.1495 |
| 66 Parietal_Inf_R 66 | 3.054 | 2 | 0.2172 | 0.4012 |
| 67 SupraMarginal_L 67 | 1.6394 | 2 | 0.4406 | 0.5552 |
| 68 SupraMarginal_R 68 | 3.4255 | 2 | 0.1804 | 0.3569 |
| 69 Angular_L 69 | 3.7251 | 2 | 0.1553 | 0.3284 |
| 70 Angular_R 70 | 6.1896 | 2 | 0.0453 | 0.1752 |
| 71 Precuneus_L 71 | 2.9493 | 2 | 0.2289 | 0.4117 |
| 72 Precuneus_R 72 | 2.4897 | 2 | 0.288 | 0.4546 |
| 73 Paracentral_Lobule_L 73 | 0.4104 | 2 | 0.8145 | 0.87 |
| 74 Paracentral_Lobule_R 74 | 0.7258 | 2 | 0.6956 | 0.7805 |
| 75 Caudate_L 75 | 1.0697 | 2 | 0.5857 | 0.6999 |
| 76 Caudate_R 76 | 0.1555 | 2 | 0.9252 | 0.9673 |
| 77 Putamen_L 77 | 4.4072 | 2 | 0.1104 | 0.2688 |
| 78 Putamen_R 78 | 8.0281 | 2 | 0.0181 | 0.1495 |
| 79 Pallidum_L 79 | 9.9099 | 2 | 0.007 | 0.1495 |
| 80 Pallidum_R 80 | 4.2938 | 2 | 0.1168 | 0.2721 |
| 83 Heschl_L 83 | 1.8858 | 2 | 0.3895 | 0.5083 |
| 84 Heschl_R 84 | 2.859 | 2 | 0.2394 | 0.4117 |
| 85 Temporal_Sup_L 85 | 2.7459 | 2 | 0.2534 | 0.4277 |
| 86 Temporal_Sup_R 86 | 4.3492 | 2 | 0.1137 | 0.2688 |
| 87 Temporal_Pole_Sup_L 87 | 7.4951 | 2 | 0.0236 | 0.1575 |
| 88 Temporal_Pole_Sup_R 88 | 2.0246 | 2 | 0.3634 | 0.4983 |
| 89 Temporal_Mid_L 89 | 3.2415 | 2 | 0.1977 | 0.3751 |
| 90 Temporal_Mid_R 90 | 0.8702 | 2 | 0.6472 | 0.7384 |
| 91 Temporal_Pole_Mid_L 91 | 2.1437 | 2 | 0.3424 | 0.4983 |
| 92 Temporal_Pole_Mid_R 92 | 5.4037 | 2 | 0.0671 | 0.2007 |
| 93 Temporal_Inf_L 93 | 0.4018 | 2 | 0.818 | 0.87 |
| 94 Temporal_Inf_R 94 | 2.0018 | 2 | 0.3675 | 0.4983 |
| 95 Cerebellum_Crus1_L 95 | 2.864 | 2 | 0.2388 | 0.4117 |
| 96 Cerebellum_Crus1_R 96 | 0.9027 | 2 | 0.6368 | 0.7369 |
| 97 Cerebellum_Crus2_L 97 | 2.4853 | 2 | 0.2886 | 0.4546 |
| 98 Cerebellum_Crus2_R 98 | 1.1371 | 2 | 0.5663 | 0.6811 |
| 99 Cerebellum_3_L 99 | 0.9761 | 2 | 0.6138 | 0.7148 |
| 100 Cerebellum_3_R 100 | 2.3338 | 2 | 0.3113 | 0.4734 |
| 101 Cerebellum_4_5_L 101 | 6.3602 | 2 | 0.0416 | 0.1752 |
| 102 Cerebellum_4_5_R 102 | 6.3873 | 2 | 0.041 | 0.1752 |
| 103 Cerebellum_6_L 103 | 3.8529 | 2 | 0.1457 | 0.3191 |
| 104 Cerebellum_6_R 104 | 6.7367 | 2 | 0.0344 | 0.1752 |
| 105 Cerebellum_7b_L 105 | 1.9 | 2 | 0.3867 | 0.5083 |
| 106 Cerebellum_7b_R 106 | 1.9446 | 2 | 0.3782 | 0.5043 |
| 107 Cerebellum_8_L 107 | 7.9594 | 2 | 0.0187 | 0.1495 |
| 108 Cerebellum_8_R 108 | 9.1658 | 2 | 0.0102 | 0.1495 |
| 109 Cerebellum_9_L 109 | 5.4597 | 2 | 0.0652 | 0.2007 |
| 110 Cerebellum_9_R 110 | 11.1619 | 2 | 0.0038 | 0.1156 |
| 111 Cerebellum_10_L 111 | 3.2858 | 2 | 0.1934 | 0.3751 |
| 112 Cerebellum_10_R 112 | 6.1114 | 2 | 0.0471 | 0.1768 |
| 113 Vermis_1_2 113 | 3.7792 | 2 | 0.1511 | 0.3272 |
| 114 Vermis_3 114 | 1.7916 | 2 | 0.4083 | 0.5253 |
| 115 Vermis_4_5 115 | 7.1665 | 2 | 0.0278 | 0.1575 |
| 116 Vermis_6 116 | 6.3771 | 2 | 0.0412 | 0.1752 |
| 117 Vermis_7 117 | 4.8127 | 2 | 0.0901 | 0.2412 |
| 118 Vermis_8 118 | 4.3438 | 2 | 0.114 | 0.2688 |
| 119 Vermis_9 119 | 6.3113 | 2 | 0.0426 | 0.1752 |
| 120 Vermis_10 120 | 2.949 | 2 | 0.2289 | 0.4117 |
| 121 Thal_AV_L 121 | 5.7664 | 2 | 0.056 | 0.1872 |
| 122 Thal_AV_R 122 | 7.3762 | 2 | 0.025 | 0.1575 |
| 123 Thal_LP_L 123 | 0.0377 | 2 | 0.9813 | 0.9957 |
| 124 Thal_LP_R 124 | 0.872 | 2 | 0.6466 | 0.7384 |
| 125 Thal_VA_L 125 | 2.0987 | 2 | 0.3502 | 0.4983 |
| 126 Thal_VA_R 126 | 3.2708 | 2 | 0.1949 | 0.3751 |
| 127 Thal_VL_L 127 | 6.1707 | 2 | 0.0457 | 0.1752 |
| 128 Thal_VL_R 128 | 7.9871 | 2 | 0.0184 | 0.1495 |
| 129 Thal_VPL_L 129 | 3.1405 | 2 | 0.208 | 0.3905 |
| 130 Thal_VPL_R 130 | 5.356 | 2 | 0.0687 | 0.2007 |
| 131 Thal_IL_L 131 | 8.7397 | 2 | 0.0127 | 0.1495 |
| 132 Thal_IL_R 132 | 4.3504 | 2 | 0.1136 | 0.2688 |
| 133 Thal_Re_L 133 | 1.1974 | 2 | 0.5495 | 0.6652 |
| 134 Thal_Re_R 134 | 13.1394 | 2 | 0.0014 | 0.0516 |
| 135 Thal_MDm_L 135 | 13.7867 | 2 | 0.001 | 0.0516 |
| 136 Thal_MDm_R 136 | 7.6439 | 2 | 0.0219 | 0.1575 |
| 137 Thal_MDl_L 137 | 8.6901 | 2 | 0.013 | 0.1495 |
| 138 Thal_MDl_R 138 | 9.1109 | 2 | 0.0105 | 0.1495 |
| 139 Thal_LGN_L 139 | 5.8053 | 2 | 0.0549 | 0.1872 |
| 140 Thal_LGN_R 140 | 3.7355 | 2 | 0.1545 | 0.3284 |
| 141 Thal_MGN_L 141 | 2.9119 | 2 | 0.2332 | 0.4117 |
| 142 Thal_MGN_R 142 | 4.7065 | 2 | 0.0951 | 0.2499 |
| 143 Thal_PuI_L 143 | 6.2947 | 2 | 0.043 | 0.1752 |
| 144 Thal_PuI_R 144 | 2.0643 | 2 | 0.3562 | 0.4983 |
| 145 Thal_PuM_L 145 | 14.6312 | 2 | 0.0007 | 0.0516 |
| 146 Thal_PuM_R 146 | 7.4418 | 2 | 0.0242 | 0.1575 |
| 147 Thal_PuA_L 147 | 8.0397 | 2 | 0.018 | 0.1495 |
| 148 Thal_PuA_R 148 | 1.4053 | 2 | 0.4953 | 0.6157 |
| 149 Thal_PuL_L 149 | 0.581 | 2 | 0.7479 | 0.824 |
| 150 Thal_PuL_R 150 | 2.5084 | 2 | 0.2853 | 0.4546 |
| 151 ACC_sub_L 151 | 0.5117 | 2 | 0.7742 | 0.843 |
| 152 ACC_sub_R 152 | 0.4994 | 2 | 0.779 | 0.8432 |
| 153 ACC_pre_L 153 | 5.4123 | 2 | 0.0668 | 0.2007 |
| 154 ACC_pre_R 154 | 4.8062 | 2 | 0.0904 | 0.2412 |
| 155 ACC_sup_L 155 | 2.0566 | 2 | 0.3576 | 0.4983 |
| 156 ACC_sup_R 156 | 3.5009 | 2 | 0.1737 | 0.3493 |
| 157 N_Acc_L 157 | 1.8455 | 2 | 0.3974 | 0.515 |
| 158 N_Acc_R 158 | 1.3358 | 2 | 0.5128 | 0.6329 |
| 159 VTA_L 159 | 2.8958 | 2 | 0.2351 | 0.4117 |
| 160 VTA_R 160 | 1.6444 | 2 | 0.4395 | 0.5552 |
| 161 SN_pc_L 161 | 1.9049 | 2 | 0.3858 | 0.5083 |
| 162 SN_pc_R 162 | 5.8639 | 2 | 0.0533 | 0.1872 |
| 163 SN_pr_L 163 | 1.017 | 2 | 0.6014 | 0.7093 |
| 164 SN_pr_R 164 | 4.4554 | 2 | 0.1078 | 0.268 |
| 165 Red_N_L 165 | 5.6414 | 2 | 0.0596 | 0.1923 |
| 166 Red_N_R 166 | 6.1915 | 2 | 0.0452 | 0.1752 |
| 167 LC_L 167 | 2.3442 | 2 | 0.3097 | 0.4734 |
| 168 LC_R 168 | 2.3644 | 2 | 0.3066 | 0.4734 |
| 169 Raphe_D 169 | 8.2408 | 2 | 0.0162 | 0.1495 |
| 170 Raphe_M 170 | 7.1334 | 2 | 0.0282 | 0.1575 |

**TABLE S11.** Comparison of long-range functional connectivity density (lrFCD) maps among healthy controls (HCs), Parkinson's disease with normal cognition (PD-NC), and Parkinson's disease with mild cognitive impairment (PD-MCI) groups based on the arterial territory template. The non-parametric Kruskal-Wallis test was used for comparisons among the three groups, with FDR-corrected significant results indicated in bold. Chi-sq: Chi-square; df: degree of freedom.

| Arterial Territories | Chi-sq | df | p value | FDR Corrected p value |
| --- | --- | --- | --- | --- |
| RACA_p | 3.4902 | 2 | 0.1746 | 0.3493 |
| RACA_m | 2.5972 | 2 | 0.2729 | 0.4484 |
| RACA_d | 1.3236 | 2 | 0.5159 | 0.6329 |
| RMCA_p | 3.2559 | 2 | 0.1963 | 0.3751 |
| RMCA_m | 5.7974 | 2 | 0.0551 | 0.1872 |
| RMCA_d | 7.3201 | 2 | 0.0257 | 0.1575 |
| RPCA_p | 8.8682 | 2 | 0.0119 | 0.1495 |
| RPCA_m | 4.4999 | 2 | 0.1054 | 0.268 |
| RPCA_d | 4.8167 | 2 | 0.09 | 0.2412 |
| LACA_p | 5.4225 | 2 | 0.0665 | 0.2007 |
| LACA_m | 6.1978 | 2 | 0.0451 | 0.1752 |
| LACA_d | 1.9832 | 2 | 0.371 | 0.4983 |
| LMCA_p | 3.9564 | 2 | 0.1383 | 0.3092 |
| LMCA_m | 7.9953 | 2 | 0.0184 | 0.1495 |
| LMCA_d | 7.3935 | 2 | 0.0248 | 0.1575 |
| LPCA_p | 8.1981 | 2 | 0.0166 | 0.1495 |
| LPCA_m | 7.5302 | 2 | 0.0232 | 0.1575 |
| LPCA_d | 2.535 | 2 | 0.2815 | 0.4546 |

Abbreviations: R, right; L, left; p, proximal territory; m, middle territory; d, distal territory; MCA, middle cerebral arteries; ACA, anterior cerebral arteries; PCA, posterior cerebral arteries.

**TABLE S12.** Comparison of amplitude of low-frequency fluctuations (ALFF) maps among healthy controls (HCs), Parkinson's disease with normal cognition (PD-NC), and Parkinson's disease with mild cognitive impairment (PD-MCI) groups based on the AAL3 atlas. The non-parametric Kruskal-Wallis test was used for comparisons among the three groups, with FDR-corrected significant results indicated in bold. Region numbering and anatomical labels were aligned with the original AAL3 atlas without modification. Chi-sq: Chi-square; df: degree of freedom.

| Label in AAL3 | Chi-sq | df | p value | FDR Corrected p value |
| --- | --- | --- | --- | --- |
| 1 Precentral_L 1 | 0.9233 | 2 | 0.6302 | 0.8283 |
| 2 Precentral_R 2 | 2.9927 | 2 | 0.2239 | 0.5671 |
| 3 Frontal_Sup_2_L 3 | 2.4088 | 2 | 0.2999 | 0.62 |
| 4 Frontal_Sup_2_R 4 | 1.8332 | 2 | 0.3999 | 0.686 |
| 5 Frontal_Mid_2_L 5 | 4.8105 | 2 | 0.0902 | 0.437 |
| 6 Frontal_Mid_2_R 6 | 1.4083 | 2 | 0.4945 | 0.7274 |
| 7 Frontal_Inf_Oper_L 7 | 0.5393 | 2 | 0.7636 | 0.8794 |
| 8 Frontal_Inf_Oper_R 8 | 3.0918 | 2 | 0.2131 | 0.5592 |
| 9 Frontal_Inf_Tri_L 9 | 2.2691 | 2 | 0.3216 | 0.6294 |
| 10 Frontal_Inf_Tri_R 10 | 1.6255 | 2 | 0.4436 | 0.7119 |
| 11 Frontal_Inf_Orb_2_L 11 | 5.6465 | 2 | 0.0594 | 0.3416 |
| 12 Frontal_Inf_Orb_2_R 12 | 1.611 | 2 | 0.4469 | 0.7119 |
| 13 Rolandic_Oper_L 13 | 3.599 | 2 | 0.1654 | 0.5072 |
| 14 Rolandic_Oper_R 14 | 0.5031 | 2 | 0.7776 | 0.8832 |
| 15 Supp_Motor_Area_L 15 | 1.8194 | 2 | 0.4026 | 0.686 |
| 16 Supp_Motor_Area_R 16 | 2.4832 | 2 | 0.2889 | 0.6194 |
| 17 Olfactory_L 17 | 5.2238 | 2 | 0.0734 | 0.3859 |
| 18 Olfactory_R 18 | 3.2385 | 2 | 0.1981 | 0.5528 |
| 19 Frontal_Sup_Medial_L 19 | 2.831 | 2 | 0.2428 | 0.5787 |
| 20 Frontal_Sup_Medial_R 20 | 0.6023 | 2 | 0.74 | 0.8728 |
| 21 Frontal_Med_Orb_L 21 | 2.1381 | 2 | 0.3433 | 0.6381 |
| 22 Frontal_Med_Orb_R 22 | 1.4279 | 2 | 0.4897 | 0.7274 |
| 23 Rectus_L 23 | 0.1368 | 2 | 0.9339 | 0.9848 |
| 24 Rectus_R 24 | 0.4061 | 2 | 0.8162 | 0.8987 |
| 25 OFCmed_L 25 | 0.0753 | 2 | 0.963 | 0.9848 |
| 26 OFCmed_R 26 | 0.609 | 2 | 0.7375 | 0.8728 |
| 27 OFCant_L 27 | 3.236 | 2 | 0.1983 | 0.5528 |
| 28 OFCant_R 28 | 0.1896 | 2 | 0.9095 | 0.973 |
| 29 OFCpost_L 29 | 0.7858 | 2 | 0.6751 | 0.8409 |
| 30 OFCpost_R 30 | 0.0915 | 2 | 0.9553 | 0.9848 |
| 31 OFClat_L 31 | 1.6343 | 2 | 0.4417 | 0.7119 |
| 32 OFClat_R 32 | 1.704 | 2 | 0.4266 | 0.7119 |
| 33 Insula_L 33 | 4.8627 | 2 | 0.0879 | 0.437 |
| 34 Insula_R 34 | 1.3007 | 2 | 0.5219 | 0.7383 |
| 37 Cingulate_Mid_L 37 | 1.133 | 2 | 0.5675 | 0.7735 |
| 38 Cingulate_Mid_R 38 | 5.7155 | 2 | 0.0574 | 0.3407 |
| 39 Cingulate_Post_L 39 | 4.1429 | 2 | 0.126 | 0.4976 |
| 40 Cingulate_Post_R 40 | 7.9531 | 2 | 0.0187 | 0.2403 |
| 41 Hippocampus_L 41 | 9.6609 | 2 | 0.008 | 0.1891 |
| 42 Hippocampus_R 42 | 3.6265 | 2 | 0.1631 | 0.5072 |
| 43 ParaHippocampal_L 43 | 7.0465 | 2 | 0.0295 | 0.28 |
| 44 ParaHippocampal_R 44 | 2.8025 | 2 | 0.2463 | 0.5787 |
| 45 Amygdala_L 45 | 6.247 | 2 | 0.044 | 0.28 |
| 46 Amygdala_R 46 | 0.7941 | 2 | 0.6723 | 0.8409 |
| 47 Calcarine_L 47 | 3.8571 | 2 | 0.1454 | 0.4976 |
| 48 Calcarine_R 48 | 3.8711 | 2 | 0.1443 | 0.4976 |
| 49 Cuneus_L 49 | 1.8682 | 2 | 0.3929 | 0.686 |
| 50 Cuneus_R 50 | 1.6147 | 2 | 0.446 | 0.7119 |
| 51 Lingual_L 51 | 8.9653 | 2 | 0.0113 | 0.1891 |
| 52 Lingual_R 52 | 9.6159 | 2 | 0.0082 | 0.1891 |
| 53 Occipital_Sup_L 53 | 4.4695 | 2 | 0.107 | 0.4895 |
| 54 Occipital_Sup_R 54 | 4.1971 | 2 | 0.1226 | 0.4976 |
| 55 Occipital_Mid_L 55 | 1.4451 | 2 | 0.4855 | 0.7274 |
| 56 Occipital_Mid_R 56 | 0.8796 | 2 | 0.6442 | 0.8297 |
| 57 Occipital_Inf_L 57 | 10.3291 | 2 | 5.70E-03 | 0.1891 |
| 58 Occipital_Inf_R 58 | 6.1003 | 2 | 0.0474 | 0.2904 |
| 59 Fusiform_L 59 | 6.7495 | 2 | 0.0342 | 0.28 |
| 60 Fusiform_R 60 | 7.4878 | 2 | 0.0237 | 0.2419 |
| 61 Postcentral_L 61 | 3.7797 | 2 | 0.1511 | 0.4976 |
| 62 Postcentral_R 62 | 4.2751 | 2 | 0.1179 | 0.4976 |
| 63 Parietal_Sup_L 63 | 2.5395 | 2 | 0.2809 | 0.6194 |
| 64 Parietal_Sup_R 64 | 0.5421 | 2 | 0.7626 | 0.8794 |
| 65 Parietal_Inf_L 65 | 6.5668 | 2 | 0.0375 | 0.28 |
| 66 Parietal_Inf_R 66 | 4.7574 | 2 | 0.0927 | 0.4372 |
| 67 SupraMarginal_L 67 | 3.9538 | 2 | 0.1385 | 0.4976 |
| 68 SupraMarginal_R 68 | 7.92 | 2 | 0.0191 | 0.2403 |
| 69 Angular_L 69 | 6.4015 | 2 | 0.0407 | 0.28 |
| 70 Angular_R 70 | 10.6487 | 2 | 0.0049 | 0.1891 |
| 71 Precuneus_L 71 | 0.1558 | 2 | 0.9251 | 0.9839 |
| 72 Precuneus_R 72 | 3.1358 | 2 | 0.2085 | 0.5592 |
| 73 Paracentral_Lobule_L 73 | 4.0785 | 2 | 0.1301 | 0.4976 |
| 74 Paracentral_Lobule_R 74 | 3.4611 | 2 | 0.1772 | 0.5094 |
| 75 Caudate_L 75 | 2.1659 | 2 | 0.3386 | 0.6381 |
| 76 Caudate_R 76 | 1.3625 | 2 | 0.506 | 0.7274 |
| 77 Putamen_L 77 | 0.0005 | 2 | 0.9998 | 0.9998 |
| 78 Putamen_R 78 | 0.098 | 2 | 0.9522 | 0.9848 |
| 79 Pallidum_L 79 | 3.0671 | 2 | 0.2158 | 0.5592 |
| 80 Pallidum_R 80 | 1.0831 | 2 | 0.5818 | 0.7872 |
| 83 Heschl_L 83 | 4.054 | 2 | 0.1317 | 0.4976 |
| 84 Heschl_R 84 | 0.9042 | 2 | 0.6363 | 0.8297 |
| 85 Temporal_Sup_L 85 | 2.7597 | 2 | 0.2516 | 0.5787 |
| 86 Temporal_Sup_R 86 | 0.6906 | 2 | 0.708 | 0.8627 |
| 87 Temporal_Pole_Sup_L 87 | 9.0225 | 2 | 0.011 | 0.1891 |
| 88 Temporal_Pole_Sup_R 88 | 3.0748 | 2 | 0.2149 | 0.5592 |
| 89 Temporal_Mid_L 89 | 2.8826 | 2 | 0.2366 | 0.5787 |
| 90 Temporal_Mid_R 90 | 0.0272 | 2 | 0.9865 | 0.9925 |
| 91 Temporal_Pole_Mid_L 91 | 3.5497 | 2 | 0.1695 | 0.5094 |
| 92 Temporal_Pole_Mid_R 92 | 1.9063 | 2 | 0.3855 | 0.686 |
| 93 Temporal_Inf_L 93 | 1.3105 | 2 | 0.5193 | 0.7383 |
| 94 Temporal_Inf_R 94 | 2.7811 | 2 | 0.2489 | 0.5787 |
| 95 Cerebellum_Crus1_L 95 | 12.3251 | 2 | 0.0021 | 0.1891 |
| 96 Cerebellum_Crus1_R 96 | 7.8655 | 2 | 0.0196 | 0.2403 |
| 97 Cerebellum_Crus2_L 97 | 6.2413 | 2 | 0.0441 | 0.28 |
| 98 Cerebellum_Crus2_R 98 | 3.8519 | 2 | 0.1457 | 0.4976 |
| 99 Cerebellum_3_L 99 | 1.5362 | 2 | 0.4639 | 0.7263 |
| 100 Cerebellum_3_R 100 | 0.4361 | 2 | 0.8041 | 0.8987 |
| 101 Cerebellum_4_5_L 101 | 0.0746 | 2 | 0.9634 | 0.9848 |
| 102 Cerebellum_4_5_R 102 | 0.7664 | 2 | 0.6817 | 0.8418 |
| 103 Cerebellum_6_L 103 | 7.4885 | 2 | 0.0237 | 0.2419 |
| 104 Cerebellum_6_R 104 | 5.3487 | 2 | 0.069 | 0.3732 |
| 105 Cerebellum_7b_L 105 | 5.3598 | 2 | 0.0686 | 0.3732 |
| 106 Cerebellum_7b_R 106 | 2.9833 | 2 | 0.225 | 0.5671 |
| 107 Cerebellum_8_L 107 | 6.7744 | 2 | 0.0338 | 0.28 |
| 108 Cerebellum_8_R 108 | 6.8835 | 2 | 0.032 | 0.28 |
| 109 Cerebellum_9_L 109 | 2.3473 | 2 | 0.3092 | 0.6288 |
| 110 Cerebellum_9_R 110 | 1.8378 | 2 | 0.399 | 0.686 |
| 111 Cerebellum_10_L 111 | 3.664 | 2 | 0.1601 | 0.5072 |
| 112 Cerebellum_10_R 112 | 1.0482 | 2 | 0.5921 | 0.7952 |
| 113 Vermis_1_2 113 | 3.6255 | 2 | 0.1632 | 0.5072 |
| 114 Vermis_3 114 | 0.5366 | 2 | 0.7647 | 0.8794 |
| 115 Vermis_4_5 115 | 3.4783 | 2 | 0.1757 | 0.5094 |
| 116 Vermis_6 116 | 11.2964 | 2 | 0.0035 | 0.1891 |
| 117 Vermis_7 117 | 0.9673 | 2 | 0.6165 | 0.822 |
| 118 Vermis_8 118 | 0.7925 | 2 | 0.6728 | 0.8409 |
| 119 Vermis_9 119 | 0.782 | 2 | 0.6764 | 0.8409 |
| 120 Vermis_10 120 | 1.1409 | 2 | 0.5653 | 0.7735 |
| 121 Thal_AV_L 121 | 1.528 | 2 | 0.4658 | 0.7263 |
| 122 Thal_AV_R 122 | 0.6719 | 2 | 0.7147 | 0.8651 |
| 123 Thal_LP_L 123 | 0.8776 | 2 | 0.6448 | 0.8297 |
| 124 Thal_LP_R 124 | 0.026 | 2 | 0.9871 | 0.9925 |
| 125 Thal_VA_L 125 | 1.4276 | 2 | 0.4898 | 0.7274 |
| 126 Thal_VA_R 126 | 0.5777 | 2 | 0.7491 | 0.8779 |
| 127 Thal_VL_L 127 | 0.3167 | 2 | 0.8536 | 0.9238 |
| 128 Thal_VL_R 128 | 0.196 | 2 | 0.9067 | 0.973 |
| 129 Thal_VPL_L 129 | 1.161 | 2 | 0.5596 | 0.7735 |
| 130 Thal_VPL_R 130 | 1.1513 | 2 | 0.5623 | 0.7735 |
| 131 Thal_IL_L 131 | 1.4469 | 2 | 0.4851 | 0.7274 |
| 132 Thal_IL_R 132 | 2.319 | 2 | 0.3136 | 0.6288 |
| 133 Thal_Re_L 133 | 0.0302 | 2 | 0.985 | 0.9925 |
| 134 Thal_Re_R 134 | 0.4052 | 2 | 0.8166 | 0.8987 |
| 135 Thal_MDm_L 135 | 0.394 | 2 | 0.8212 | 0.8987 |
| 136 Thal_MDm_R 136 | 0.12 | 2 | 0.9418 | 0.9848 |
| 137 Thal_MDl_L 137 | 1.4474 | 2 | 0.485 | 0.7274 |
| 138 Thal_MDl_R 138 | 2.1465 | 2 | 0.3419 | 0.6381 |
| 139 Thal_LGN_L 139 | 2.4562 | 2 | 0.2929 | 0.6194 |
| 140 Thal_LGN_R 140 | 1.6469 | 2 | 0.4389 | 0.7119 |
| 141 Thal_MGN_L 141 | 1.861 | 2 | 0.3944 | 0.686 |
| 142 Thal_MGN_R 142 | 1.4022 | 2 | 0.496 | 0.7274 |
| 143 Thal_PuI_L 143 | 3.804 | 2 | 0.1493 | 0.4976 |
| 144 Thal_PuI_R 144 | 0.5117 | 2 | 0.7743 | 0.8832 |
| 145 Thal_PuM_L 145 | 0.9392 | 2 | 0.6253 | 0.8277 |
| 146 Thal_PuM_R 146 | 0.3837 | 2 | 0.8254 | 0.8987 |
| 147 Thal_PuA_L 147 | 2.6101 | 2 | 0.2712 | 0.616 |
| 148 Thal_PuA_R 148 | 1.6471 | 2 | 0.4389 | 0.7119 |
| 149 Thal_PuL_L 149 | 0.8282 | 2 | 0.6609 | 0.8409 |
| 150 Thal_PuL_R 150 | 0.4465 | 2 | 0.7999 | 0.8987 |
| 151 ACC_sub_L 151 | 2.3126 | 2 | 0.3146 | 0.6288 |
| 152 ACC_sub_R 152 | 4.4316 | 2 | 0.1091 | 0.4895 |
| 153 ACC_pre_L 153 | 1.6024 | 2 | 0.4488 | 0.7119 |
| 154 ACC_pre_R 154 | 3.1398 | 2 | 0.2081 | 0.5592 |
| 155 ACC_sup_L 155 | 2.5135 | 2 | 0.2846 | 0.6194 |
| 156 ACC_sup_R 156 | 6.2604 | 2 | 0.0437 | 0.28 |
| 157 N_Acc_L 157 | 3.9187 | 2 | 0.1409 | 0.4976 |
| 158 N_Acc_R 158 | 1.3683 | 2 | 0.5045 | 0.7274 |
| 159 VTA_L 159 | 1.2863 | 2 | 0.5256 | 0.7383 |
| 160 VTA_R 160 | 0.6429 | 2 | 0.7251 | 0.872 |
| 161 SN_pc_L 161 | 2.2117 | 2 | 0.3309 | 0.6343 |
| 162 SN_pc_R 162 | 2.1038 | 2 | 0.3493 | 0.6426 |
| 163 SN_pr_L 163 | 2.2925 | 2 | 0.3178 | 0.6288 |
| 164 SN_pr_R 164 | 2.8994 | 2 | 0.2346 | 0.5787 |
| 165 Red_N_L 165 | 3.809 | 2 | 0.1489 | 0.4976 |
| 166 Red_N_R 166 | 6.3643 | 2 | 0.0415 | 0.28 |
| 167 LC_L 167 | 1.9077 | 2 | 0.3853 | 0.686 |
| 168 LC_R 168 | 0.616 | 2 | 0.7349 | 0.8728 |
| 169 Raphe_D 169 | 4.0244 | 2 | 0.1337 | 0.4976 |
| 170 Raphe_M 170 | 2.5525 | 2 | 0.2791 | 0.6194 |

**TABLE S13.** Comparison of amplitude of low-frequency fluctuations (ALFF) maps among healthy controls (HCs), Parkinson's disease with normal cognition (PD-NC), and Parkinson's disease with mild cognitive impairment (PD-MCI) groups based on the arterial territory template. The non-parametric Kruskal-Wallis test was used for comparisons among the three groups, with FDR-corrected significant results indicated in bold. Chi-sq: Chi-square; df: degree of freedom.

| Arterial Territories | Chi-sq | df | p value | FDR Corrected p value |
| --- | --- | --- | --- | --- |
| RACA_p | 2.2165 | 2 | 0.3301 | 0.6343 |
| RACA_m | 1.3918 | 2 | 0.4986 | 0.7274 |
| RACA_d | 2.014 | 2 | 0.3653 | 0.6655 |
| RMCA_p | 2.4717 | 2 | 0.2906 | 0.6194 |
| RMCA_m | 4.8141 | 2 | 0.0901 | 0.437 |
| RMCA_d | 6.6801 | 2 | 0.0354 | 0.28 |
| RPCA_p | 9.0636 | 2 | 0.0108 | 0.1891 |
| RPCA_m | 7.6777 | 2 | 0.0215 | 0.2419 |
| RPCA_d | 3.5035 | 2 | 0.1735 | 0.5094 |
| LACA_p | 2.427 | 2 | 0.2972 | 0.62 |
| LACA_m | 0.7044 | 2 | 0.7031 | 0.8625 |
| LACA_d | 0.4087 | 2 | 0.8152 | 0.8987 |
| LMCA_p | 8.0635 | 2 | 0.0177 | 0.2403 |
| LMCA_m | 0.1019 | 2 | 0.9503 | 0.9848 |
| LMCA_d | 3.7752 | 2 | 0.1514 | 0.4976 |
| LPCA_p | **19.4974** | **2** | **0.0001** | **0.0107** |
| LPCA_m | 9.1743 | 2 | 0.0102 | 0.1891 |
| LPCA_d | 2.8355 | 2 | 0.2423 | 0.5787 |

Abbreviations: R, right; L, left; p, proximal territory; m, middle territory; d, distal territory; MCA, middle cerebral arteries; ACA, anterior cerebral arteries; PCA, posterior cerebral arteries.
